# Host matters: coral reef fish species show distinct skin microbiome responses to upwelling-driven environmental changes

**DOI:** 10.1186/s12866-026-05036-1

**Published:** 2026-04-21

**Authors:** Laura L. Lardinois, Natasha A. Hinojosa, Helio Quintero-Arrieta, Andrew J. Sellers, Matthieu Leray, Rowan D. H. Barrett

**Affiliations:** 1 Department of Biology, McGill University, Montreal, QC H3A 0G4 Canada; 2 Smithsonian Tropical Research Institute, Balboa, Ancon, Republic of Panama; 3 Department of Ecology and Evolutionary Biology, University of California Santa Cruz, Santa Cruz, CA USA; 4 School of Biological Sciences, Swire Institute of Marine Science, The University of Hong Kong, SAR, Hong Kong, China

**Keywords:** Skin microbiome, Coral reef fishes, Host-microbe interaction, Host-microbe interactions, Environmental change, Marine biodiversity, Panama

## Abstract

**Background:**

Disentangling the drivers structuring microbiomes can help predict organisms’ responses to rapid environmental change. However, despite microbial communities being important for both host and environmental health, large gaps remain in our understanding of how host-associated microbiomes are structured and respond to different stimuli, especially in marine environments. Here, we leverage a seasonal upwelling gradient in Panama’s Tropical Eastern Pacific, investigating how the diversity and composition of ten coral reef fish species’ skin microbiomes compare between regions and seasons that experience different environmental conditions.

**Results:**

Fish skin microbiomes varied greatly within and among host species and were distinct from the microbiomes of the surrounding seawater. All species had diverse skin microbiomes, with a dominance of Proteobacteria (65%), Bacteroidota (13%), and Cyanobacteria (6%). Host species and trophic group played a greater role in determining fish skin microbiome structure than seasonal and regional environmental variation, despite water microbiomes responding strongly to both season and region. Nevertheless, three out of five trophic groups: the herbivores, carnivores, and planktivore, also displayed significant changes in their microbiomes during upwelling, albeit to a lesser extent than water samples, whereas the invertivores’ and omnivore’s microbiomes did not change significantly. We performed differential abundance (DA) analyses on these fish and compared microbial taxa that changed between seasons and regions in fish versus water samples. While water communities had thousands of significant DA taxa, fish had around 40 times fewer (*n* = 17 to 73) and only shared 47 DA taxa with the water samples. Differences between these microbial communities likely arise from both host selection via fishes’ immune system and the skin mucus serving as an environmental filter. However, neither host-associated nor environmental predictors fully explained the variation in microbiome composition, highlighting its complexity.

**Conclusions:**

Our results show how ecological differences between host species may elicit distinct microbiome responses to environmental changes, with potential cascading effects on ecosystem dynamics under global climate change. Further characterization of marine microbial communities, as well as additional physicochemical and host-related parameters, will be key to monitoring and predicting how these communities will respond to the increasingly rapid and widespread environmental changes our oceans are facing.

**Supplementary Information:**

The online version contains supplementary material available at 10.1186/s12866-026-05036-1.

## Background

Microbes are the unseen majority on Earth. Trillions of microbes; including bacteria, archaea, viruses, and fungi, grow on and inside living organisms, forming their unique microbiome [1, 2]. These microbial communities establish a dynamic relationship with their animal hosts, responding to factors like the host’s biology, diet, and the surrounding environment [3]. Some members of the microbiome fulfil essential functions such as providing nutrients, regulating metabolism, and protecting against pathogens, whereas others can be pathogenic or parasitic [4, 5]. Furthermore, microbiomes not only influence host traits and the surrounding environment, but also contribute an “extended genetic repertoire” and can rapidly respond to their environment - via changes in community composition, gene expression, and rapid evolution, with important implications for their hosts [6–8].

At the ecosystem scale, microbes play crucial roles in nutrient cycling, animal and plant health, agriculture, and aquaculture [9]. The cumulative impacts of climate change, including ocean warming and decreasing oxygen levels, are threatening the biosphere, especially the marine ecosystems that make up over 70% of the planet [10, 11]. These stressors not only impact animal hosts directly, but also disrupt the balance between hosts and their microbiomes, yet the full extent of their impacts on microbiomes are unknown [9, 12]. Emerging research from coral reefs, which are among the most biodiverse and threatened ecosystems, shows cause for concern: for instance, butterflyfish gut microbiomes were altered on degraded reefs, and reef fish gut microbiomes changed when exposed to nutrient pollution in the laboratory [13, 14]. Untangling the complex factors that influence the myriads of resident and transient microbial associates is key to understanding how organisms will respond in an era of rapid environmental change.

Microbes that colonize the surface of hosts’ skin live at the interface between hosts and the external environment, providing an ideal system to study the interactions between hosts, microbes, and responses to environmental change. Furthermore, skin microbiomes play a crucial role in host health, serving as the first line of defence against pathogens, which is especially important for marine organisms in constant contact with microbes in the surrounding water [5, 15]. Despite their importance for host health, much less is known about the composition of the skin microbiomes of wild organisms, including coral reef fishes, compared to the gut microbiome, and the degree to which these communities respond to environmental changes. Existing work has concentrated on a small number of Caribbean reef fishes sampled in one location over a short period, making it hard to generalize findings across the wider diversity of reef fishes and trophic groups (cleaner gobies [16–18]; damselfishes [19]). Other studies had broader taxonomic coverage, but relied on very small per-species sample sizes (avg. *n* = 3) and single collection events [20, 21]. More recently, data collected for two fish species, three coral species, and plankton across the Pacific revealed that microbiomes not only differed between hosts, but also responded more strongly to geographic distance than local environmental conditions (e.g. salinity and temperature) [22]. No study of coral reef fish microbiomes has integrated spatial, temporal and taxonomic dimensions, limiting our ability to understand how host identity and environmental variability interact to shape these microbial communities. This knowledge gap is particularly important given concerns about the potential vulnerability of reef-associated microbiomes to environmental change and biodiversity loss given the unique communities associated with each host species [20]. Our study provides a novel characterization of fish skin microbiomes in multiple host species across different regions and seasons, with populations experiencing different degrees of recurring environmental changes driven by seasonal upwelling.

**Fig. 1:**
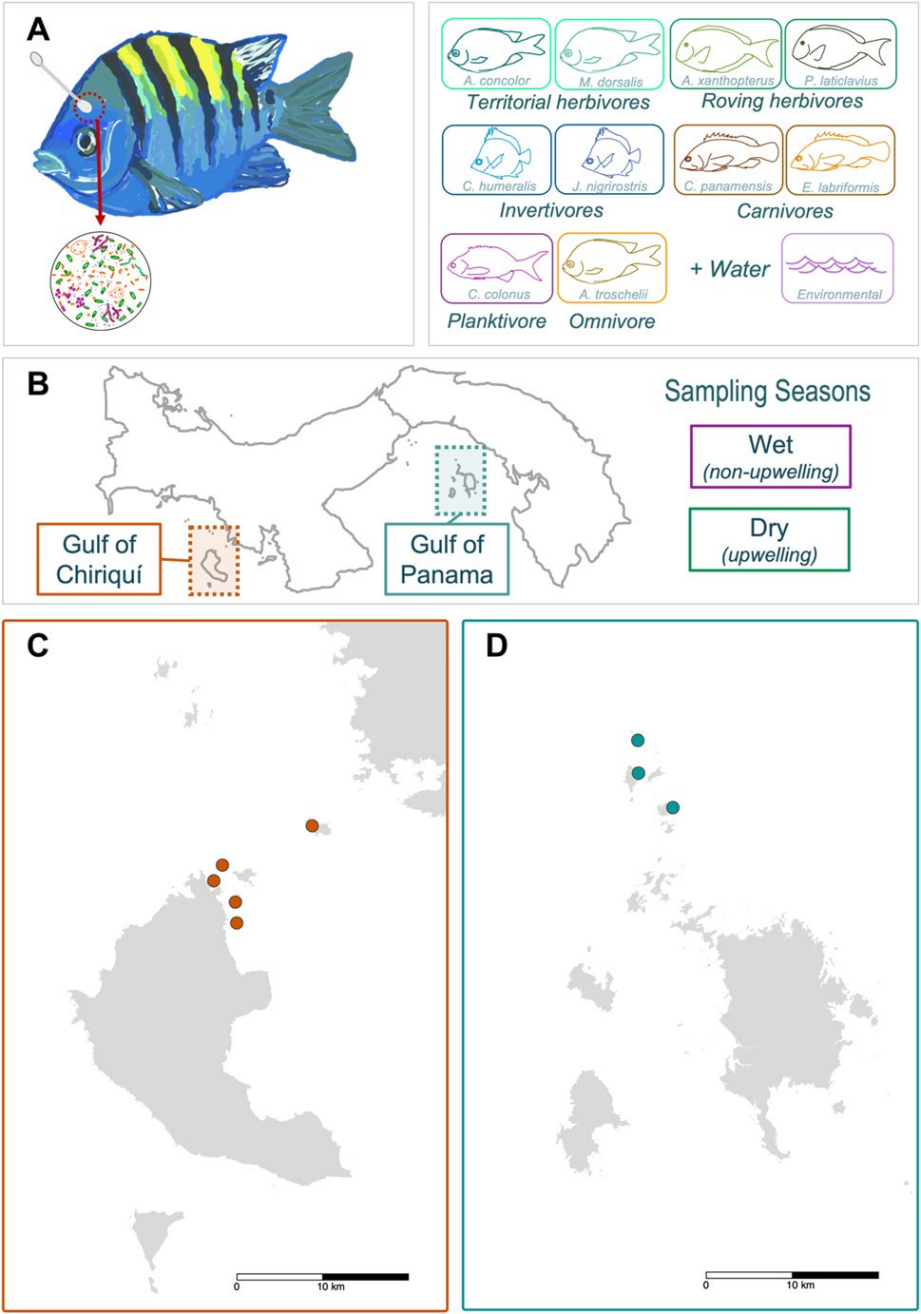
Study design and sampling sites in Panama’s Tropical Eastern Pacific. (**A**) Study species: Skin swabs were collected from the upper dorsal side of 10 fish species from 6 trophic groups: territorial herbivores (turquoise), roving herbivores (dark green), invertivores (blue), carnivores (brown), a planktivore (dark purple), and an omnivore (orange). Water samples (light purple) were collected for comparison. (**B**) Map of Panama and sampling regions: Samples were collected from two gulfs in the TEP of Panama: (**C**) the Gulf of Chiriquí (dark orange), to the west, and (**D**) the Gulf of Panama (teal), to the east, during the wet (Oct-Nov) and dry (Mar-Apr) seasons. Map source file: M. Solano (2022), fish illustrations and icons hand-drawn by L. Lardinois

**Fig. 2:**
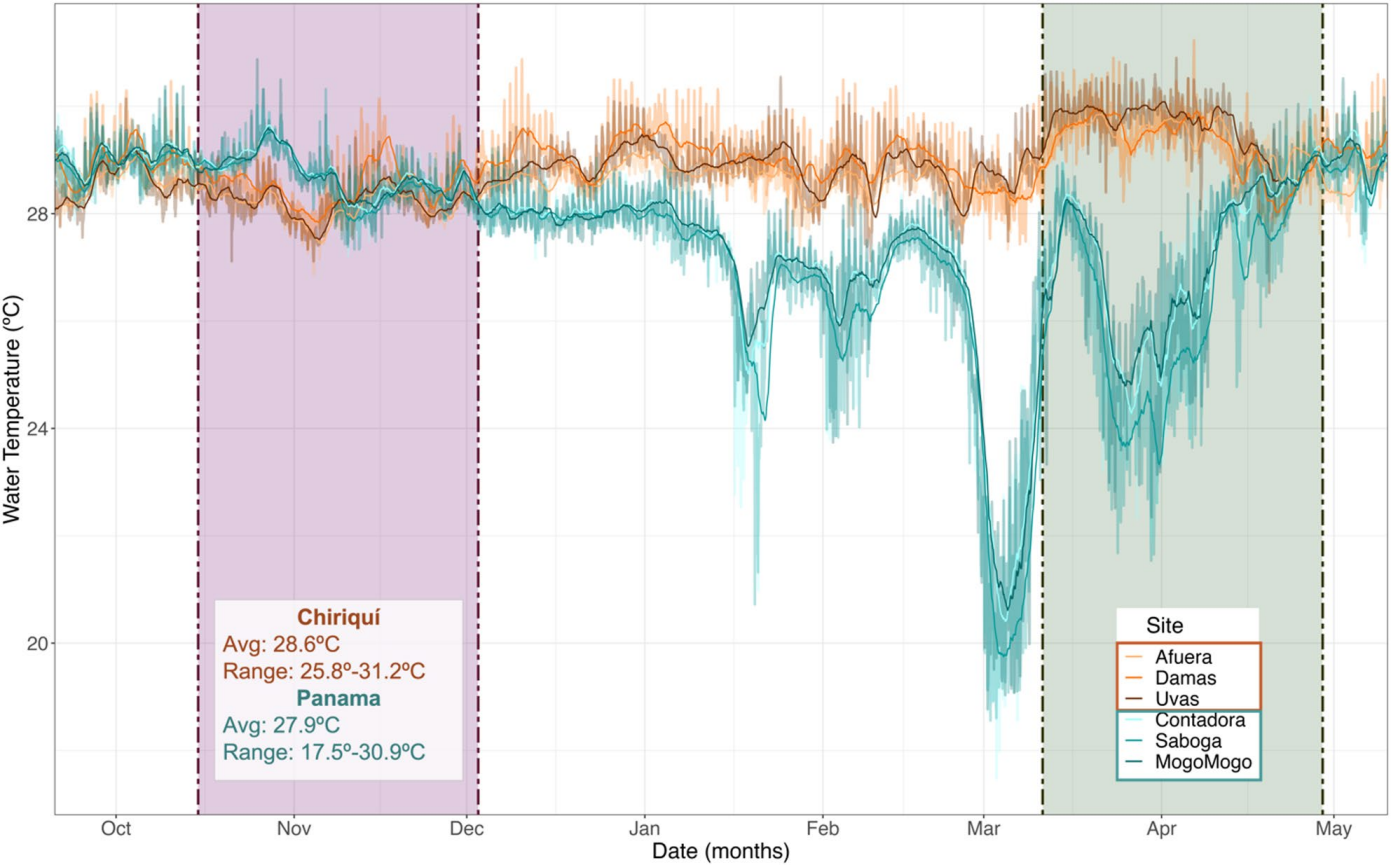
Water temperature in the TEP. Temperatures (ºC) in the Gulf of Chiriquí (orange) and Gulf of Panama (teal). Shaded bars: sampling in wet (purple) and dry (green) seasons. Dry season sampling began after water temperatures dropped in the Gulf of Panama, indicative of upwelling. Simple moving average (48 h avg. temp) overlaid over raw temperature values

We leverage natural environmental variation created by seasonal upwelling in Panama’s Tropical Eastern Pacific (Fig. 1) to test how environmental changes influence the diversity and composition of fish host-associated microbiomes. Within the Tropical Eastern Pacific (TEP), the Gulf of Panama experiences upwelling during the dry season (*Jan-Apr*), when trade winds displace surface water, causing cold, low-oxygen, nutrient-rich water to rise from the depths [23, 24]. To the west, the Cordillera Central mountains block these trade winds, weakening upwelling in the nearby Gulf of Chiriquí (Fig. 2) [24, 25]. During the wet season (*Apr-Dec*), conditions in the two gulfs return to being similar [25]. This phenomenon is an ideal natural experiment to explore how environmental changes impact skin microbiomes and whether these microbiomes are most strongly influenced by environmental factors or host-associated traits [26]. Both regions harbour similar communities of coral reef fishes with many ecologically and economically important species 27].

In this study, we used 16S rRNA marker gene sequencing to quantify how the skin microbiome responds to changing environmental conditions across regions and seasons in 10 species of reef fishes spanning a range of trophic groups (Table S2). We compared host-associated microbiomes to microbial communities in the surrounding water, and whether these communities respond in similar ways to upwelling conditions. We hypothesized that, if hosts are the principal determinants of microbial communities, the skin microbiomes on fish belonging to the same species would be most similar in composition and relative abundance, with host species and host-associated factors such as trophic group playing a greater role than region or season (H1). Alternatively, if skin microbiomes are primarily influenced by environmental factors, microbiomes from the same region and season would be most similar across species (H2). Furthermore, we would expect greater similarity between skin and water microbiomes, as well as parallel responses to upwelling conditions. Understanding the factors that shape these complex microbial communities in a natural system is essential to better predict the effects of stressors under global climate change.

## Methods

### Study area and sample collection

We collected fish samples from two island archipelagos in the TEP: Coiba in the Gulf of Chiriquí, which does not experience strong upwelling, and Las Perlas in the Gulf of Panama, which experiences strong upwelling during the dry season (Fig. 1). Within each archipelago, we sampled from several sites to ensure that we captured a representative sample of fish host-associated microbiomes (Table S1). We conducted two rounds of sampling in each region across 2021–22: near the end of the wet season (non-upwelling; Oct. 15 – Dec. 3, 2021), and at the end of the dry season (upwelling; Mar. 11 – Apr. 29, 2022). Environmental data, including water temperature and dissolved oxygen (DO), were collected with HOBO and miniDOT data loggers, respectively, in both gulfs (Fig. 2; Fig. S1).

We selected 10 abundant, ecologically important reef-dwelling fish species in five trophic groups: four herbivores (roving and territorial), two invertivores, two carnivores, a planktivore, and an omnivore, representing four families: Acanthuridae (surgeonfishes), Chaetodontidae (butterflyfishes), Pomacentridae (damselfishes), and Serranidae (groupers) (Fig. 1; Table S2). We replicated fish species within each trophic group wherever possible and included representatives from the same family belonging to different trophic groups (see Pomacentridae and Serranidae; Table S2). We acknowledge there is still overlap between fish families and trophic groups, limiting our ability to differentiate between familial and trophic level effects. Given that prior work has shown high inter- and intra-specific microbiome variability [21], we aimed to collect 10 adult fish per species from each region and season. We collected morphological data, including sex, length, mass, and gonad mass, as sex, size, body condition, and reproductive status could all have effects on the microbiome [28–30]. We excluded fish that expressed juvenile characteristics (size and/or colouration), and focused on individuals of the same size classes from each region, to control for microbiome shifts that are known to occur across ontogeny [31, 32].

We used freediving and spearfishing (1–15 m depth) to collect the fish following protocols approved by the Smithsonian Tropical Research Institute’s IACUC (SI-22047). Using gloved hands, we rubbed a sterile swab across the upper dorsal side of each fish 3–5 times, circumventing bodily fluids and avoiding contact with the fisher’s hand. After swabbing, fish were euthanized using the rapid chilling method, then kept on ice until we returned from the field. We placed swabs in cryotubes, immediately flash-froze them in liquid nitrogen, then stored them at −80ºC until DNA extraction. Field control swabs (exposed to air in the field) were collected at each site and season (*n* = 10 GoC, *n* = 9 GoP), following the same protocol for flash-freezing and storing alongside the fish samples. Additionally, we collected 2-L water samples above the reef at each site with Whirl–Pak bags, aiming to collect around 5 samples per region and season. To collect these samples, a freediver dove down to approximately 15–30 cm above the reef near our fish sampling sites, opened a sealed 2L Whirl–Pak bag, swam forward to fill it, then rolled the lip shut to seal the bag at depth before bringing it to the surface. Water samples were kept on ice for transport to our field station, run through Millipore™ 0.22 um MCE membranes within 2–3 h of collection using a vacuum filtration system, and membranes were stored at −80ºC until DNA extraction. Filtration blanks were also collected in the field by running 2L of DI water over a clean 0.22 um MCE membrane from the same pack, in between two of our environmental water samples. All filtration equipment was pre-sterilized and separate clean funnels were used for each sample to prevent any cross-contamination risk.

### DNA extraction & sequencing

We extracted fish skin microbial DNA using the ZymoBIOMICS 96 well DNA kit (four 96-well plates in total), following the manufacturer’s instructions. Briefly, we snipped swabs containing fish skin mucus, as well as field control swabs, with sterile scissors and placed the cotton tip in 750 uL Zymo DNA/RNA Shield (instead of lysis solution) in bead-beating tubes, before continuing with the standard protocol. Environmental (water) DNA was extracted from membranes using the Qiagen DNeasy PowerSoil Kit. Membranes from filtration blanks were extracted at the same time. We amplified the V4 region of the 16S ribosomal RNA gene (16S rRNA) using primers 515 F and 806R adapted for Illumina sequencing, following a modified version of the Earth Microbiome Project 16S protocol [33] (PCR conditions: Table S3). Phased primers – one set per plate – were used to increase sample library complexity and augment sequencing quality. Negative PCR controls and extraction controls were included in each plate. Filtration blanks were run on gels alongside the samples and technical controls, checked to ensure no detectable contamination (no visible band or smear), but excluded from downstream steps to reduce sequencing costs. Index PCR was performed to attach unique barcodes, then all four plates: 384 samples including all fish skin samples, field controls, extraction, and PCR controls, were pooled for sequencing on the Illumina MiSeq sequencing platform of the Smithsonian Tropical Research Institute's (STRI) Naos facilities in Panama (paired end 250 bp with v2 chemistry). Water samples were PCR-amplified independently following identical protocols as the fish samples and run on a separate sequencing run to avoid potential contamination. To minimize inter-run differences, water samples were run on the same sequencer with the same settings within a month, and all library and sample prep was done by the same researcher.

### Sequence data processing

Sequenced libraries were demultiplexed using the MiSeq Reporter Software. We trimmed these libraries with Cutadapt (v 4.1) to remove primers and adaptors before reading them into R version 4.2.2 for downstream analyses following the DADA2 pipeline (tutorial v 1.16) [34]. Reads from the fish and water samples were processed independently and, unless otherwise indicated, analyses were run on the two datasets separately. Trimmed sequence reads are available at EMBL-EBI under accession number [PRJEB104461]. We plotted the quality profiles of the forward and reverse reads to visualize read quality prior to setting truncation thresholds for filtering and trimming (set at 220 (Fwd) and 180 (Rev) base pairs). We then calculated error rates, dereplicated, inferred amplicon sequence variants (using the pseudo-pool method to capture rare ASVs) and merged forward and reverse reads. Next, we removed chimeras (method = pooled) and assigned taxonomy using the SILVA reference taxonomy (v. 138.1) [35].

Contaminants were detected with the ‘isContaminant’ function from the `decontam` R package, using the prevalence method, which identifies contaminants based on their increased prevalence in control samples [36]. A total of 35 and 10 contaminant sequences were detected in the fish and water datasets, respectively (*see supplementary files S1 & S2 for complete decontam outputs*). Following contaminant detection, all contaminant sequences, along with ASVs assigned to chloroplasts, mitochondria, eukaryotes, or unassigned at the phylum level were removed, as were control samples. This brought us to 4,418,389 reads and 23,440 ASVs (range: 1-48,663 sequences/sample) across 359 fish samples. We then filtered out potentially spurious ASVs found in less than two samples. Additionally, samples with fewer that 1,000 reads were removed, as these likely amplified poorly and do not fully capture the diversity of the fish skin microbiome, bringing us down to 341 fish samples and 6551 ASVs in the cleaned dataset (Table S4A). By comparison, the water sample dataset had 1,507,994 total reads, corresponding to 2,712 ASVs across 19 samples (range: 42,156-99,992 sequences/sample; Table S4B). To test for the influence of uneven sequencing depths on patterns of community composition, we conducted all downstream analyses with both an unrarefied and a rarefied version of the datasets.

The rarefied dataset was assembled by computing rarefaction curves and normalizing the reads to equal library sizes based on these curves. We first used mirl() from the `mirlyn` package [37] to repeatedly rarefy samples to 2500 reads (10 times), following recent publications on best practices for normalizing microbiome data [38, 39]. Then, after confirming that sample richness remained consistent across iterations, we rarefied the dataset to 2500 reads using the rarefy_even_depth() function from the `phyloseq` package [40]. The rarefied fish dataset included 6,493 ASVs across 279 samples (62 samples fell below the 2500 read threshold and were dropped; Table S4A). The water dataset was rarefied to two different sequencing depths: first to the lowest sequencing depth across all water samples (40,000) to compare water microbiomes between regions and seasons, then to the smaller rarefaction threshold of the fish dataset (2500), for direct comparisons between the fish and water microbiomes. Given that results were consistent between unrarefied and rarefied datasets, we present the unrarefied data in the main text, with the rarefied results available in the supplementary materials. To calculate phylogenetic diversity, we created a maximum likelihood phylogenetic tree (GTR + G + I) for all ASVs, using `DECIPHER` (v 2.26.0) and `phangorn` (v 2.11.1) packages for multiple alignment and tree construction, respectively [34, 41, 42].

### Microbiome analyses

#### Assessing alpha diversity (diversity within hosts)

**Fig. 3:**
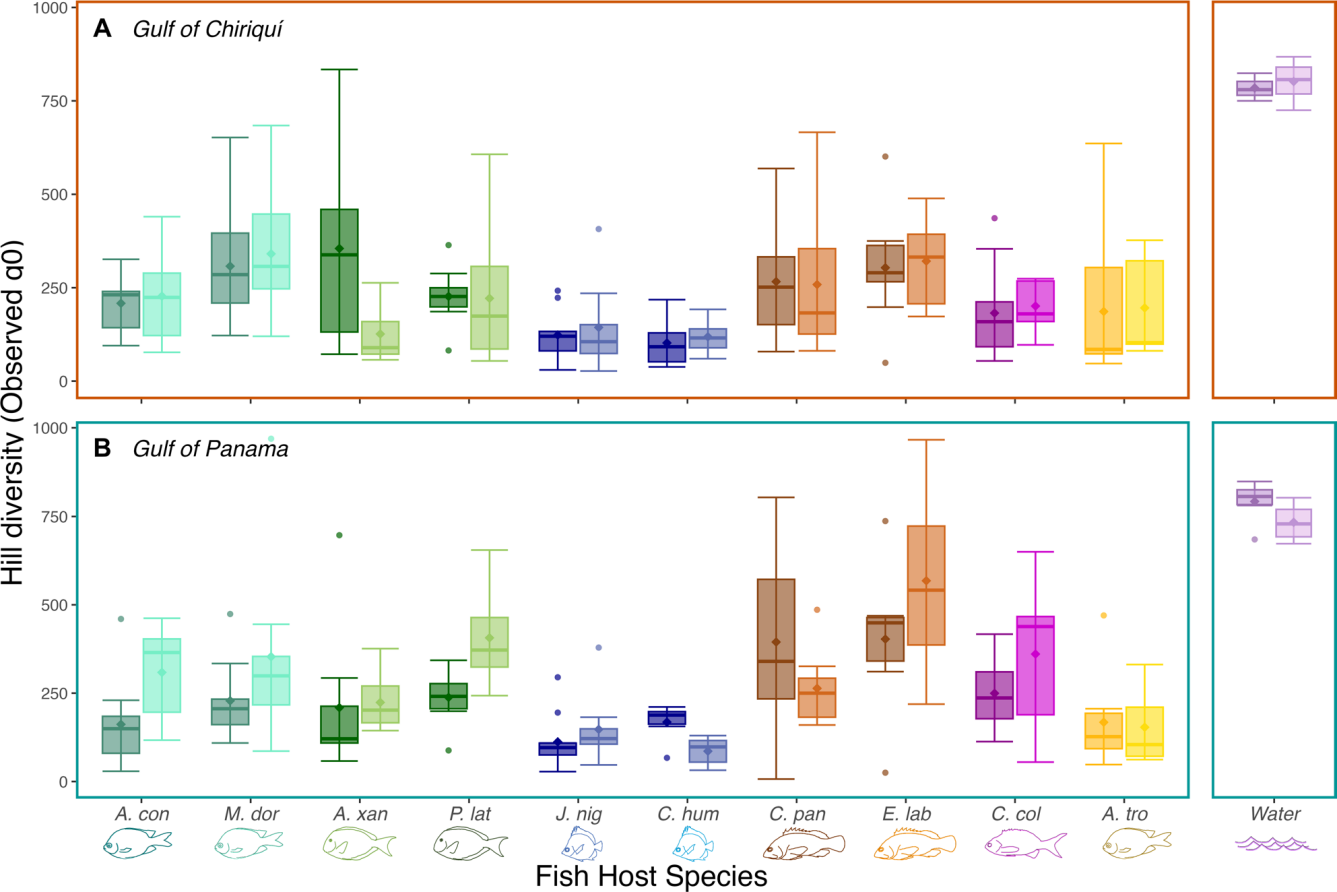
***Alpha diversity—observed richness by gulf. ***Boxplots of observed richness for each host species, split by gulf: (**A**) Gulf of Chiriquí (orange box) and (**B**) Gulf of Panama (teal box). In each panel, samples are split by season; left (dark shade) = wet season, right (light shade) = dry season. Bar colours and fish icons indicate trophic groups* and study species, respectively (see Fig. 1). Right-most panels: water samples. *Trophic group colour key: turquoise = territorial herbivores, dark green = roving herbivores, blue = invertivores, brown = carnivores, dark purple = planktivore, orange = omnivore, light purple = water samples).

We leveraged Hill numbers, a trio of complementary metrics: observed (species richness, which provides higher weight to rare taxa), Shannon exponential (uses a logarithmic scale, balancing rare and common taxa), and Simpson’s multiplicative inverse (emphasizing common taxa), to measure alpha diversity in our samples [34, 43] (Fig. 3, S2). Prior to running Shannon and Simpson’s indices, we transformed the data to relative abundances using the transform() wrapper function from the `microbiome` package to convert the data stored in the phyloseq object to a compositional format [44]. Together, these metrics allowed us to visualize within-sample diversity in the fish species, across sampling regions and seasons. We then ran non-parametric Kruskal-Wallis tests with post hoc Dunn tests to compare alpha diversity among our fish species, and across sampling regions and seasons [13]. Additional diversity, evenness, and dominance metrics are compiled in supplementary materials (Table S5).

#### Assessing beta diversity (diversity between hosts)

**Fig. 4:**
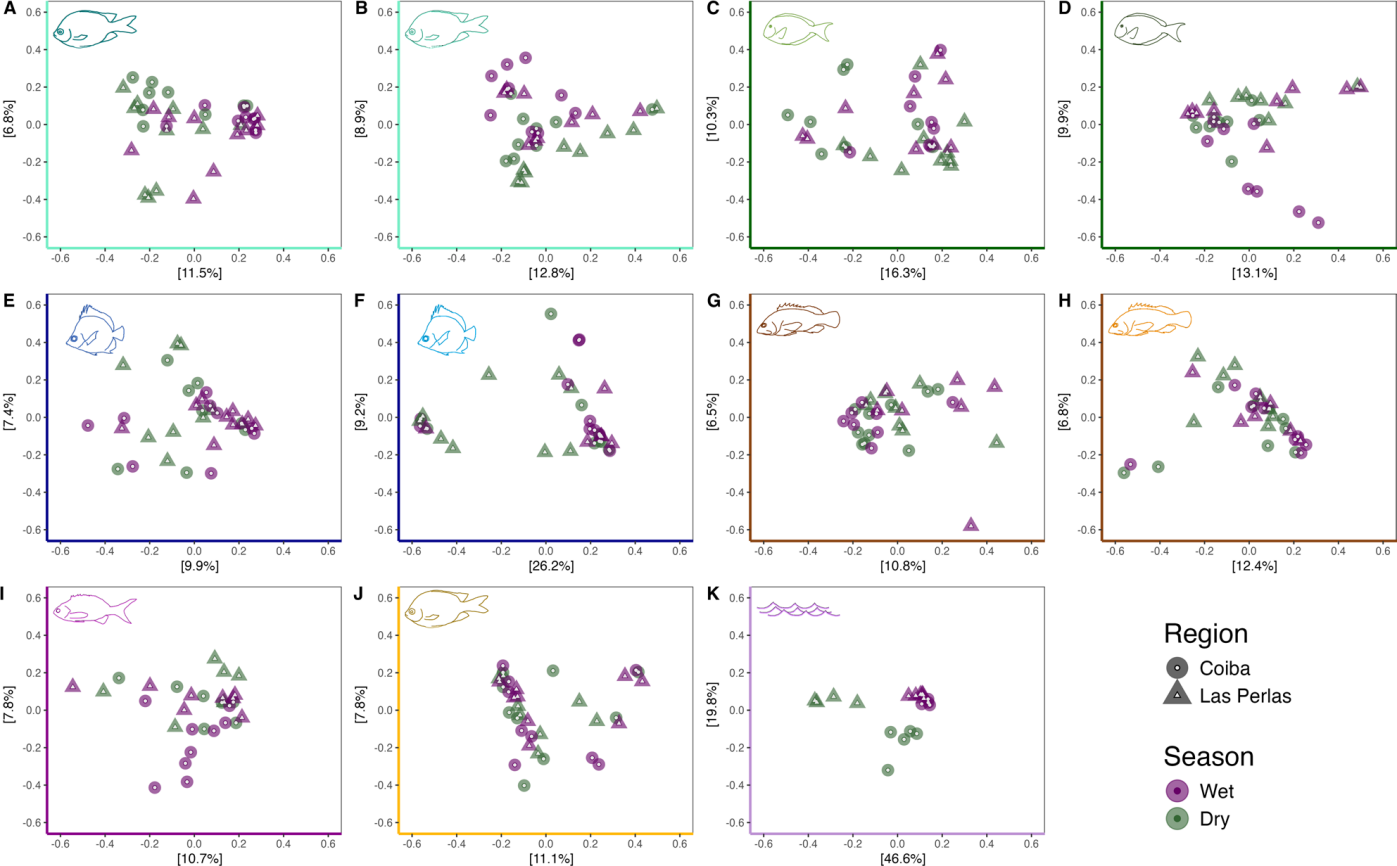
*Dissimilarity between microbial communities*. PCoA plots based on Bray–Curtis dissimilarities between communities. (**A**-**J**) Fish skin microbiomes across regions (circle: Gulf of Chiriquí; triangle: Gulf of Panama) and seasons (purple: wet, green: dry), split by host species. Axis colours and icons indicate trophic groups* and study species, respectively (see Fig. 1). (**K**) Water microbiome. *Trophic group colour key: turquoise = territorial herbivores, dark green = roving herbivores, blue = invertivores, brown = carnivores, dark purple = planktivore, orange = omnivore, light purple = water samples)

**Fig. 5:**
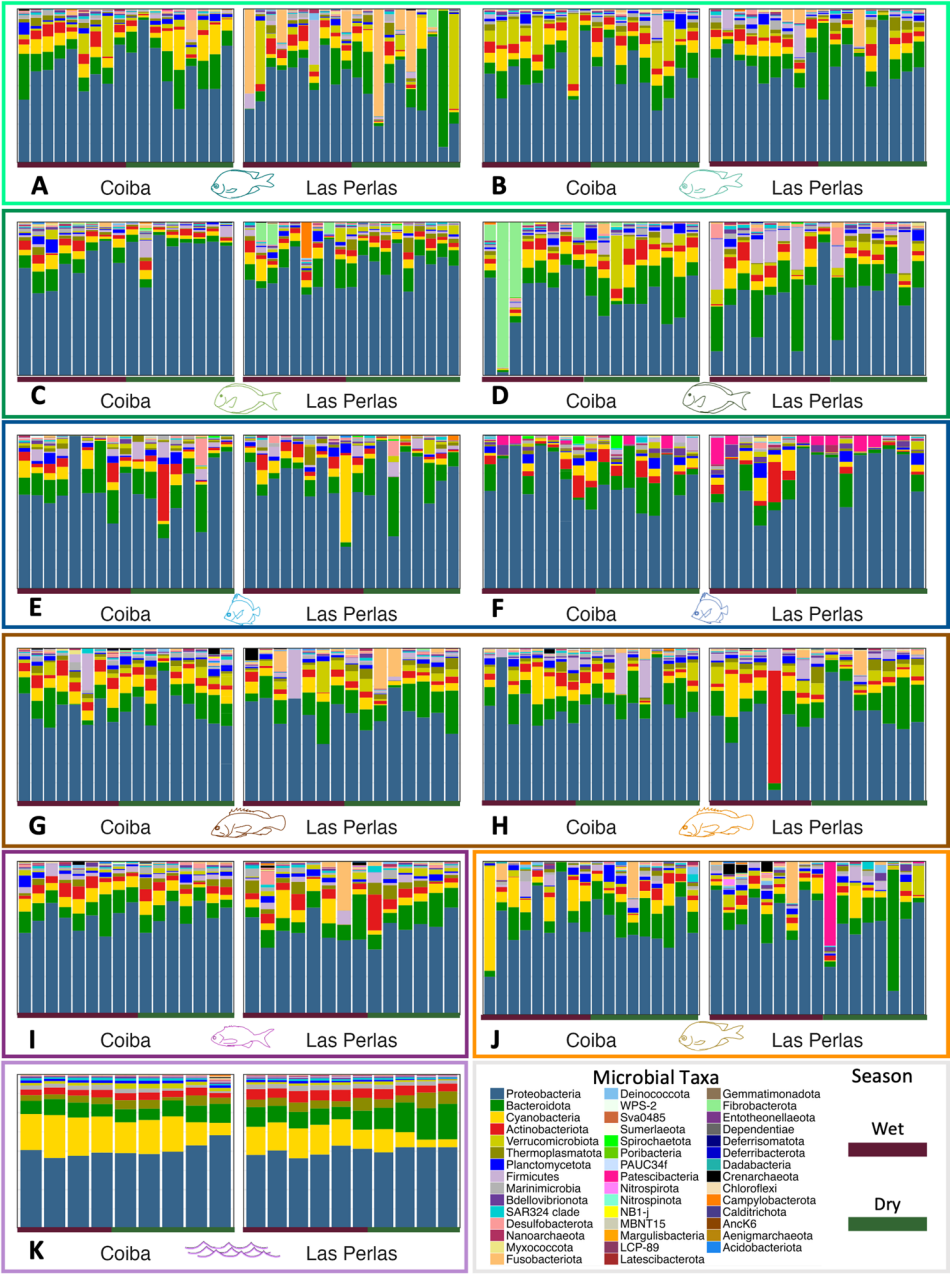
*Microbial relative abundances*. Bar plots of phylum-level microbiome composition, split by gulf (left: Coiba - Gulf of Chiriquí, right: Las Perlas - Gulf of Panama). (**A**-**J**) Each bar represents a single fishes’ microbiome. Panels are arranged by trophic group (coloured boxes: turquoise = territorial herbivores, dark green = roving herbivores, blue = invertivores, brown = carnivores, dark purple = planktivore, orange = omnivore, light purple = water samples), fish icons denote species (see Fig. 1), and coloured lines along the bottom of each panel mark seasons (purple = wet, green = dry). (**K**) Water microbiome community

We used a range of metrics to tease apart the influence of rare versus common ASVs (Jaccard and Bray Curtis), and the phylogenetic relatedness of ASVs (unweighted UniFrac) on patterns of community dissimilarity. Total Sum Scaling (TSS) was applied to both the non-rarefied and rarefied datasets prior to running statistical analyses that could be sensitive to sequencing depth (e.g., Bray–Curtis), using the phyloseq function transform_sample_counts() and applying the following function: x/sum(x) (TSS). This function normalizes ASV counts across each sample, returning (compositional) relative abundances sample-by-sample, which work well with distance metrics such as Bray–Curtis. We ran individual permutational multivariate analyses of variance (PERMANOVA) to test for the effect of host species, host trophic group, sampling region, and sampling season. We then ran additional PERMANOVAs testing for interactions between these key variables, constraining permutations to within a trophic group, given the nestedness of species within trophic groups. Results using Bray–Curtis dissimilarity are presented in the main text. All other metrics are in the supplementary materials (Table S7). Ordinations (PCoA and NMDS) were used to compare microbial community (dis)similarity (Figs. 4, S3, S4A & S4B). We visualized microbial relative abundance across host species, regions, and seasons with stacked bar plots (Figs. 5, S5). Then, we ran an Indicator Analysis using the indval function from the `labdsv` package, following the methods outlined in [13] to define a core fish skin microbiome. This method calculates an indicator value for each ASV and incorporates both relative frequency and abundance of ASVs in the metric (*see supplementary materials: part 6*). All analysis and visualization scripts are available on GitHub (https://github.com/lardinois21/RRR_Fish_Microbiome_16S.git) and are archived on Zenodo (10.5281/zenodo.17739194).

#### Differentially abundant taxa between seasons and regions

We assessed which microbial taxa were differentially abundant between seasons and regions using differential abundance analyses. DESeq2 (differential gene expression analysis based on the negative binomial distribution) and MaAsLin2 (microbiome multivariable association with linear models) were among the best performing DA tests, particularly for low sample sizes, in a recent comparison [45–47]. Initial tests on our water dataset showed that the DESeq2 and MaAsLin2 results were comparable (*supp. mat.: MaAsLin2*), but we ran DESeq2 on the fish dataset given the flexibility provided by the “contrasts” argument for testing pairwise comparisons. Differential abundance tests were only run for fish species with significant differences in microbiome composition based on the PERMANOVA results. Un-normalized counts were used, as DESeq2 corrects for differences in library size. Prior to running the tests, we filtered out taxa present in less than 10% of samples in each dataset, as rare taxa can impact the model assumptions and false discovery rate (FDR) penalty applied [48]. We specified contrasts to test four pairwise comparisons: (1) upwelling-independent seasonal changes in the Gulf of Chiriquí (Gulf of Chiriquí wet season vs. dry season), (2) upwelling-associated seasonal changes in the Gulf of Panama (Gulf of Panama dry season vs. wet season), (3) “baseline” inter-region differences (Gulf of Panama wet season vs. Gulf of Chiriquí wet season, when environmental conditions are similar between the two gulfs), and (4) inter-region differences during upwelling (Gulf of Panama dry season vs. Gulf of Chiriquí dry season) (Table S8, Figs. S6-S14). Finally, we ran each of the significant DA taxa in the fish datasets against the DA taxa from the water dataset to distinguish between changes in taxa that may be occurring in the surrounding environment and carrying over to the fish skin, versus taxa that are changing independently in the fish, irrespective of the surrounding water microbial community (Table S9). For contrasts with sufficient shared taxa (i.e., filtering for fishes with > 4 shared ASVs per region-season comparison), we calculated Pearson’s correlations on the log2-fold change in ASV abundance in the fish versus water datasets to assess whether the direction and magnitude of change matched between environmental and host-associated communities (Fig. S15).

## Results

### Strong seasonal shifts in temperature and dissolved oxygen in the Gulf of Panama

As expected for upwelling, during the dry season water temperatures dropped sharply in the Gulf of Panama beginning in late February (min: 17.5ºC) whereas they remained stable throughout the year (~ 29ºC) in the Gulf of Chiriquí (Fig. 2). This was accompanied by a drop in dissolved oxygen concentrations in the Gulf of Panama, from an average of 6.55 mg/l (96% DO saturation), down to as low as 0.86 mg/l (11% sat.; Fig. S1). In contrast, DO concentrations varied widely daily in the Gulf of Chiriquí but exhibited no obvious seasonal patterns (range: 0.9–19.92 mg/l (14–249% sat.), avg.: 6.45 mg/l (99% sat.); Fig. S1).

### The fish skin microbiome is highly variable, dominated by key phyla, and distinct from seawater

Fish skin microbiomes were highly diverse, varied greatly within and among host species, yet remained distinct from microbial communities in the surrounding water (Figs. 4 and 5). At the phylum level, the dominant members of the fish skin microbiome were Proteobacteria (averaging 65% relative abundance across species), Bacteroidota (13%), Cyanobacteria (6%), Actinobacteriota (4%), and Verrucomicrobiota (3%, Fig. 5). Water samples also contained these phyla, however, their relative abundances differed, with less Proteobacteria (54%) and more Cyanobacteria (18%, Fig. 5K). Dominant phyla were shared between fish skin and water samples, yet certain phyla were only found in fish: Latescibacteria, MBNT15, Deferrisomatota, Deferribacterota, LCP-89, and Sumerlaeota. Only four phyla were unique to the water samples: AncK6, Nitrospinota, Aenigmarchaeota, and Poribacteria. Some microbial phyla were highly represented in multiple samples of a given fish species, for instance, Fusobacteriota in *Abudefduf concolor* (Fig. 5A), Fibrobacterota in *Prionurus laticlavius* (Fig. 5D), and Patescibacteria in *Chaetodon humeralis* (Fig. 5E). Firmicutes appeared in many fish samples, irrespective of species, with its abundance varying considerably to make up over 25% of some samples while being almost completely absent from others. The core microbiome analysis returned 12 ‘core’ taxa found across all fish samples, belonging to classes Alphaproteobacteria (4), Gammaproteobacteria (7), and Deinococci (1) (Fig. S16). These ‘core’ ASVs ranged in abundance and frequency: for instance, a *Pseudomonas* bacterium (ASV 785), was found in 313 samples, while ASV 1076, belonging to the genus *Tepidimonas*, was detected in 106 samples. Note that although prokaryotic nomenclature has recently been revised [49], we refer to the phylum names in the SILVA database (v. 138.1) used for taxonomic assignment [35].

### Microbial richness increases in dry season for herbivores and planktivores in the Gulf of Panama

When analysing the full dataset, there were no significant differences in fish skin microbiome alpha diversity across regions and seasons for any of the three metrics (observed, Shannon exponential, and Simpson’s multiplicative inverse; Kruskal–Wallis with BH correction; *p* > 0.05), although the Gulf of Panama tended to have higher observed richness during the dry season (upwelling) compared to wet season samples from both the Gulf of Panama and the Gulf of Chiriquí ( *p* = 0.12 and 0.17, respectively). However, pairwise comparisons showed significant differences in alpha diversity between many fish species, with the most pronounced differences in carnivores, which had higher diversity, and invertivores, which had lower diversity (Figs. 3, S2; Tables S5, S6). Splitting the dataset by species, we see a slight but non-significant increase in alpha diversity during the dry season (upwelling) in the Gulf of Panama across all four herbivores and the planktivore, whereas no clear regional or seasonal pattern is visible in the other species and trophic groups (Fig. 3). Similar patterns were seen across all Hill numbers, and differences tended to be more pronounced in observed richness, suggesting these differences are driven in part by rare ASVs. In comparison, for the water samples, Simpson’s multiplicative inverse was significant (*p* = 0.019), suggesting that shifts in common taxa in the Gulf of Panama during the dry season cause these microbial communities to become significantly different from those in both the dry and wet season in the Gulf of Chiriquí.

### Fish skin microbiome beta diversity is primarily driven by host species and trophic group

Host species and trophic group had a greater influence on fish skin microbiome structure than environmental factors such as sampling region or season. Although each of these factors are significant, across the full dataset host species explains 7.9% of the variation in microbiome composition, trophic group explains 4.5%, whereas region and season only explain 1.4% and 1.3%, respectively (PERMANOVA – Bray–Curtis, species: R^2^ = 0.079, *p* < 0.001; trophic group: R^2^ = 0.045, *p* < 0.001; region: R^2^ = 0.014, *p* < 0.001; season: R^2^ = 0.013, *p* = 0.001, Table S7). We also found significant interactions between many combinations of predictors, including region and season (R^2^ = 0.008, *p* < 0.001, Bray–Curtis); diet and region (R^2^ = 0.017, *p* = 0.002), and diet, species, region, and site (R^2^ = 0.108, *p* = 0.051) (Table S7). Combinations of predictors better explained microbiome composition than a single predictor. Other potential explanatory factors such as fish sex and size either had no significant effect on the skin microbiome (sex), or minimal explanatory power (length and mass) (Table S7). Importantly, even when combining the best predictors we measured, over 66% of the variation in fish skin microbiome remained unexplained.

Unconstrained ordinations (PCoA and NMDS; Bray–Curtis dissimilarity) did not reveal consistent clustering in the fish microbiome: samples from different regions and seasons generally overlapped in two-dimensional space, likely due to the low level of variance explained by these factors (Figs. 4, S3, S4). Given the importance of host species for skin microbiome community structure, we tested the effect of environmental factors on each species separately. We found consistent patterns for fish belonging to the same trophic group. Across trophic groups, hosts generally fell into two categories: ones for which neither region nor season were significant (the invertivores and the omnivore), and ones where both region and season had a significant impact on the skin microbiome (the herbivores and carnivores, the planktivore, and the water) (Fig. 4, Table S7). Furthermore, fish in the latter category differed in whether there was a significant interaction between region and season: the territorial herbivores and one carnivore (*E. labriformis*) had no interaction whereas the roving herbivores, the other carnivore (*C. panamensis*), the planktivore, and the water had significant interactions between season and region.

When comparing the microbial beta diversity results obtained using Bray–Curtis dissimilarity to those from Jaccard and UniFrac, all three indices generally showed similar trends (i.e., significant differences for the same host species, regions, and seasons) in our microbiome data, but differed in the species-by-species analyses. For instance, we found more significant differences between regions and seasons across host species for Bray–Curtis and Jaccard compared to UniFrac, signalling that there are changes in relative abundance and presence/absence of taxa, but that at the phylogenetic level, the communities do not change as much (Table S7). In other words, while individual microbial taxa inhabiting the skin may change between host species, and vary across both season and region, they are likely being replaced by similar taxa, such that from a phylogenetic perspective, the microbial communities are not that different.

Compared to the fish, region and season influenced the water microbial communities to a much greater extent, as reflected by the higher percent variance (PERMANOVA; Bray–Curtis, season: R^2^ = 0.313, *p* < 0.001; region: R^2^ = 0.190, *p* = 0.005, Table S7). Furthermore, as would be expected given that only one region experiences strong seasonal upwelling, we found significant interactions between sampling season and region (R^2^ = 0.15, *p* < 0.001). These effects were equally apparent in our ordinations, where samples from the Gulf of Panama and the Gulf of Chiriquí clustered tightly together during the wet season and broke apart into two separate, distinct clusters in the dry (upwelling) season (Fig. 4K).

### More differentially abundant taxa between regions and seasons in water than in fish

After analysing the differences in microbial community composition across seasons, regions, and hosts, we leveraged differential abundance analyses to better understand which microbial taxa might be driving these differences. Among our water samples, 524 taxa (of 1,298 filtered taxa) differed significantly in abundance between the Gulf of Panama wet and dry season samples (DESeq2; Table S8, Fig. S7). Of these, 318 (24%) were enriched (log-fold change > 0) and 206 (16%) decreased in the wet relative to the dry season. There were fewer DA taxa across gulfs during the wet season (116), suggesting that seasonal differences outweigh regional ones in the water microbiomes (Table S8, Fig. S6). Compared to the water, fish had fewer significant DA taxa, principally Proteobacteria, with a few Bacteroidota, Cyanobacteria, and Verrucomicrobiota (Table S8). Given the fish skin microbiome’s diversity, the same 10% filter prior to running DESeq2 resulted in only 16–24% of each fish host’s taxa being retained for the analysis (mean: 658 taxa). While certain fish, such as *M. dorsalis* and *P. laticlavius* (two herbivores) followed expected patterns, with the greatest number of DA taxa between the wet and dry seasons in the Gulf of Panama (Table S8; Figs. S9, S11), the majority did not follow this pattern. Instead, DA taxa occurred randomly across the four pairwise region-season comparisons, with many DA taxa in certain host species while others had few (range: 17–73; Table S8). Certain taxa, such as the cyanobacterium *Prochlorococcus* sp*.* and the proteobacterium *Ascidiaceihabitans* sp., were differentially abundant in the upwelling vs non-upwelling comparison (Gulf of Panama) across several host species and water samples.

### Overlap in differentially abundant taxa between fish and water microbiomes

Approximately a quarter of ASVs identified as DA in fish were also differentially abundant in the water, although the majority of these shared ASVs came from the two herbivorous host species mentioned above: *M. dorsalis* and *P. laticlavius*, which shared 52% and 48% of their DA taxa with water, respectively (range of shared DA across all species: 9–52%; Tables S8, S9). 47 unique microbial taxa were significantly differentially abundant in both water and at least one host fishes’ skin (Tables S8, S9). Twenty of these appeared multiple times (DA across various host species and/or in several region-season comparisons; again, this overlap was principally seen in both *M. dorsalis* and *P. laticlavius*; Table S9). Most of the shared taxa were Proteobacteria, including *Ascidiaceihabitans sp*. and several members of the SAR86 clade. In all but two ASVs, the direction of change matched between the fish and water samples, such that if taxa were enriched in water in the Gulf of Panama during the dry season, for instance, they would also be enriched in the fish (Table S9; Fig. S15). Furthermore, most shared DA taxa were found within one of two comparisons: Gulf of Panama wet vs. dry (*n* = 38), and Gulf of Panama dry vs. Gulf of Chiriquí dry (*n* = 36). These two comparisons capture upwelling-driven seasonal differences in the Gulf of Panama (Table S8). Diving further into these patterns, the direction and magnitude of changes observed in these shared ASVs followed an almost one-to-one relationship with the water samples in two of the three species (*M. dorsalis* and *P. laticlavius*) in the Gulf of Panama wet vs. dry and Gulf of Panama vs. Gulf of Chiriquí dry season comparisons (r = 0.84–0.96, *p* < 0.001; Pearson’s correlation; *Fig. S15*). On the other hand, no correlation was found for *A. xanthopterus*, which shared 10 ASVs with the water samples in the Gulf of Panama vs. Gulf of Chiriquí dry season comparison (r = 0.3, *p* = 0.392).

## Discussion

### Host and trophic group effects

This study sought to quantify how the skin microbiomes of reef fish species inhabiting two gulfs of the TEP are structured and respond to contrasting seasonal change across two regions. Host species identity, in conjunction with trophic group, was the strongest predictor of both alpha and beta diversity in reef fishes’ skin microbiomes, in line with H1. This also aligns with other work that has found unique host-associated skin microbiomes relative to the surrounding water, suggesting that fishes’ skin selects for a distinct microbial community, rather than taking up microbes indiscriminately from the surrounding water [20, 21, 50, 51]. While trophic group and/or diet are known to strongly influence the gut microbiome, our work is one of the first to show that these factors also play an important role for fish skin microbiome composition [52, 53]. Importantly, host species remains significant when including diet as a predictor, which precludes the observed host species effect from simply being an artefact of a given species’ dietary preferences [51]. Additional support for this host effect comes from paired microbiome and host genotyping work in four sympatric Serrasalmidae (piranha) species, which showed that skin microbiomes and host genotypes covaried significantly both within and across species [54]. Conserved relationships between hosts and their skin microbiomes could, in turn, lead to phylosymbiosis, such that microbial communities mirror their hosts’ phylogenies [55]. However, given the high degree of intra-specific variability in these fishes’ microbiomes, we caution against recent attempts to detect phylosymbiosis in teleosts using datasets with low species-level replication [56, 57].

Understanding how hosts may shape their skin microbiomes requires a closer look at the skin’s surface. From a microbial perspective, the skin mucosa provides a unique niche space of gelatinous mucin [58]. However, for microbes to establish communities in the fish skin requires that they evade – or coexist with – enzymes, immune proteins, antimicrobial peptides, and other components of the hosts’ innate immune system, in addition to other competing microbial taxa [59]. Hosts share similar physical traits with their conspecifics, including the skin, scales, and mucosal layer, forming a similar environment that selects for certain microbes [60]. Additionally, certain immune defences in teleost skin are highly conserved, whereas others appear to be clade or species-specific, or develop throughout an individual host’s lifetime, thus imposing distinct constraints on potential colonizers [60, 61]. Shared host traits and innate immunity would help account for the significant explanatory power of host species in determining the fish skin microbiome.

### Environmental effects

Upwelling systems are distributed globally and have wide-ranging impacts on the productivity of marine ecosystems [62]. How microbial communities in the water column respond to upwelling has received considerable attention in other upwelling areas, including the California Current, the Benguela Current, and Tongoy Bay [63–65]. However, our study is the first to assess these phenomena in the TEP of Panama, in which we observe large shifts in water microbial communities during upwelling, particularly in the Gulf of Panama, where upwelling is strongest (Figs. 4, S3, S4, S6). Water microbiomes were more similar between gulfs during the wet season (non-upwelling) than they were within a gulf across seasons, indicating that seasonal turnover of microbial taxa outweighs fixed differences between gulfs (Fig. 4, Table S8). Interestingly, while the greatest number of differentially abundant taxa in the water communities were, as expected, seen in the Gulf of Panama during the dry season, when there is strong upwelling, we also saw a clear signal of seasonal change in the Gulf of Chiriquí during the dry season, despite no strong changes in temperature nor dissolved oxygen during this time. This may be due to other seasonal changes in coastal hydrology, such as surface runoff, altering water microbiomes during the wet season, or be linked to a localized upwelling hotspot in the Gulf of Chiriquí [25, 66].

Furthermore, this is the first study to look at the effects of upwelling-driven environmental changes on both water and vertebrate host-associated microbiomes. Compared to the surrounding seawater, significant seasonal changes were only detected in a subset of our host species’ microbiomes and were of lesser magnitude. Host-associated microbiomes had around 40 times fewer significant differentially abundant (DA) taxa across region-season comparisons than water (Table S8). Among these, hosts shared ~ 26% of their DA taxa with water samples: this shared portion may have been picked up directly from the surrounding environment, and thus reflects the large seasonal changes seen in the water communities. Most host-associated DA taxa, however, responded to environmental changes independently. The shared taxa included several members of the SAR86 clade, an abundant and widespread marine group, which have previously been shown to vary geographically and seasonally [67]. Similarly, the cyanobacterium *Prochlorococcus sp.*, which decreased in abundance in the Gulf of Panama during upwelling, is one of the most abundant marine photosynthetic organisms and has both shallow and deep-adapted forms [68]. In this case, upwelling may be replacing the common, shallow-water strains with taxa from deeper waters. Two host species (*M. dorsalis* and *P. laticlavius*) were more sensitive to these environmental microbiome shifts, with around half of their DA taxa matching ones in the water communities and fluctuating seasonally in the same way (Fig. S15).

Seasonal changes in temperature and dissolved oxygen (Figs. 2, S1, S4B) associated with upwelling likely played a role in the differences seen in the fish skin microbiomes, as both variables (alongside salinity and pH) correlated with shifts in microbiome diversity in a recent review of the few studies to date that have reported DO and temperature data alongside microbiome results for saltwater fishes’ skin [69]. Hosts whose skin microbiomes were altered by seasonal upwelling – namely, the herbivores, carnivores, and planktivore – may be more susceptible to future environmental changes. However, without further functional information, we cannot determine whether the community-level changes we observe are harmful or helpful to the host. Microbes demonstrate high levels of phenotypic plasticity and can thrive under diverse conditions, allowing them to respond to changing environmental conditions [70, 71]. Thus, rapid shifts in microbial community composition and metabolic activity could help the host, if the microbiome is altered in such a way that it continues to provide necessary services [72]. On the other hand, disruptions in the existing microbiome could favour the establishment of pathogenic taxa with detrimental effects. The differences in responses to seasonal environmental changes between seawater and host-associated microbial communities highlight the need for further study of the two in tandem, to better understand how host-microbe relationships may be impacted by environmental changes in different host species, populations, and habitats at risk.

### Microbiome complexity and residual variation

Individual fishes’ traits, life histories, and adaptive immune responses, in combination with microbe-microbe interactions and stochastic changes in the community due to drift, may help explain the 66% variation that was neither captured by host species nor season or region [70]. When considering this residual variation, it is worth noting that, while we focus here on host species as a predictor, we also assessed the effects of factors such as sex and size, which have previously been shown to impact fish gut microbiomes [28–30]. We found no significant effect of sex and only limited effects of size, suggesting that – at least in adult fishes of these species—these traits are not major determinants of microbiome structure. Genetic diversity within host species may be another factor contributing to host-associated microbiome variation, as reef fishes often display both high genetic diversity and low genetic differentiation, even across distant reef sites [73, 74]. Thus, future work pairing host genomics and microbiome analyses could serve the dual purpose of furthering our understanding of these reef fishes’ population genetic structure and how it may affect their associated microbial communities. Furthermore, once microbes successfully reach the fish skin, potential colonizers must compete to establish themselves in this limited niche space. Priority effects, including niche pre-emption (the first taxa to arrive take over), niche modifications (microbial taxa altering their environment to favour their own growth), and microbial antagonism (competition for limited resources and antimicrobial defences), play an important role in shaping host-associated microbial communities [75]. While such microbial assembly mechanisms are difficult to measure, especially in wild populations, repeated sampling of individuals prior to, during, and following upwelling may aid to further disentangle individual, species, regional, and seasonal effects in microbial community assembly.

## Conclusions

Our findings suggest that host-associated microbiome responses to future environmental changes will likely be host-specific, with certain species or taxonomic groups responding more strongly. Being associated with a host imposes constraints on the microbial communities that can form, via host immunity and environmental filtering, which acts as a buffer against the fluctuations seen in water microbial communities. These results raise additional questions: *(1)* Why do certain hosts’ microbiomes respond more strongly to environmental changes and is this an indicator of vulnerability under future climate change? *(2)* Which microbiome functional shifts are occurring, and what are their repercussions on host and ecosystem health? *(3)* Are there additional deterministic factors that may address the unexplained variation in these host-associated microbial communities? Further sampling of marine organisms’ microbiomes with greater replication within and across species, families, and trophic groups would provide crucial insights into this first question. Techniques such as metabolomics and metatranscriptomics can be leveraged to determine functional shifts in microbial communities. Finally, monitoring additional physicochemical and host-related parameters, including sub-species level population structure via genomics research, may help further disentangle microbial community dynamics. Given the global scale of the environmental changes our oceans are facing, it is imperative that we build on our nascent understanding of the role microbes play in host and ecosystem health.

## Supplementary Information

Supplementary Material 1. Master supplementary file. Includes details on study design, additional environmental data, alpha and beta diversity results, differential abundance analyses, and core microbiome analyses.

Supplementary Material 2. Contaminant detection with decontam.

Supplementary Material 3. Supplementary data tables. Includes full list of alpha diversity metrics (Table S5), PERMANOVA results (Table S7), all differentially abundant taxa (Table S8), shared DA taxa between fish and water (Table S9), and core taxa identified by the indicator analysis (Table S10).

## Data Availability

Additional methods, metadata, figures, and tables are available as supplementary materials. Raw sequence reads have been deposited to the European Nucleotide Archive (ENA) at EMBL-EBI under accession number [PRJEB104461]. Code for analyses and figures are available as maintained versions on GitHub (https://github.com/lardinois21/RRR_Fish_Microbiome_16S.git) and have been archived on Zenodo https://doi.org/10.5281/zenodo.17739194).
