# Supplementary material for: Host matters: coral reef fish species show distinct skin microbiome responses to upwelling-driven environmental changes
Source: BMC Microbiol. 2026 Apr 21;26:527. doi: 10.1186/s12866-026-05036-1 (PMC13238074; doi:10.1186/s12866-026-05036-1)
Supplement: Supplementary file 1 — Supplementary Material 1. Master supplementary file. Includes details on study design, additional environmental data, alpha and beta diversity results, differential abundance analyses, and core microbiome analyses. [file 12866_2026_5036_MOESM1_ESM.docx]

**Supplementary Materials - *Host matters: coral reef fish species show distinct skin microbiome responses to upwelling-driven environmental changes***

**Table of Contents**

[Part 1: General Study Design 3](#_Toc223556155)

[Table S1: GPS coordinates & metadata for sampling sites. 3](#_Toc223556156)

[Table S2: Study species – Tropical reef fish of Panama 3](#_Toc223556157)

[Table S3: PCR conditions for 16S rRNA gene amplification 4](#_Toc223556158)

[Table S4A: Fish Sample Sizes. 4](#_Toc223556159)

[Table S4B: Water Sample Sizes. 5](#_Toc223556160)

[Supplementary files S1 & S2: contaminant detection with decontam. 5](#_Toc223556161)

[Part 2: Environmental Parameters 6](#_Toc223556162)

[Figure S1: Water temperature and dissolved oxygen in Panama’s Tropical Eastern Pacific. 6](#_Toc223556163)

[Part 3: Alpha diversity metrics 7](#_Toc223556164)

[Figure S2: Hill numbers by host species. 7](#_Toc223556165)

[Table S5: Alpha diversity metrics 8](#_Toc223556166)

[Table S6: Alpha diversity pairwise comparisons between species. 8](#_Toc223556167)

[Part 4: Beta diversity metrics 9](#_Toc223556168)

[Table S7: PERMANOVA results. 9](#_Toc223556169)

[Figure S3: Dissimilarity between microbial communities - PCoA: rarefied to 2500 reads. 9](#_Toc223556170)

[Figure S4A: Dissimilarity between microbial communities - NMDS. 10](#_Toc223556171)

[Figure S4B: Dissimilarity between microbial communities with covariates - NMDS. 11](#_Toc223556172)

[Figure S5: Relative abundances of skin microbial phyla – rarefied to 2500 reads 12](#_Toc223556173)

[Part 5: Differential abundance analyses 13](#_Toc223556174)

[MaAsLin2: 13](#_Toc223556175)

[Table S8: Differentially abundant taxa between regions and seasons for fish and water microbiomes. 14](#_Toc223556176)

[Table S9: Significant DA taxa shared between fish and water microbiomes. 14](#_Toc223556177)

[Figure S6: Water taxa differential abundance between regions and seasons. 15](#_Toc223556178)

[Figure S7: Water taxa differential abundance between seasons in Las Perlas. 16](#_Toc223556179)

[Figure S8: A. concolor taxa differential abundance in between seasons and regions. 17](#_Toc223556180)

[Figure S9: M. dorsalis taxa differential abundance between seasons and regions. 18](#_Toc223556181)

[Figure S10: A. xanthopterus taxa differential abundance between seasons and regions. 19](#_Toc223556182)

[Figure S11: P. laticlavius taxa differential abundance in between seasons and regions. 20](#_Toc223556183)

[Figure S12: C. panamensis taxa differential abundance in between seasons and regions: DESeq2. 21](#_Toc223556184)

[Figure S13: E. labriformis taxa differential abundance in between seasons and regions: DESeq2. 22](#_Toc223556185)

[Figure S14: C. colonus taxa differential abundance in between seasons and regions: DESeq2. 23](#_Toc223556186)

[Figure S15: Comparison of changes in DA taxa between fish and water samples. 24](#_Toc223556187)

[Part 6: Core microbiome analyses 24](#_Toc223556188)

[Indicator Analysis: 24](#_Toc223556189)

[Table S10: Species-by-species core microbiomes. 25](#_Toc223556190)

[Figure S16: Core microbial taxa and their distribution across host species. 26](#_Toc223556191)

Supplementary Materials

# **Part 1:** General Study Design

## Table S1: GPS coordinates & metadata for sampling sites.

| **Sampling site** | **Gulf** | **Coordinates** |
| --- | --- | --- |
| Machete | Gulf of Chiriquí | N7º38’17.4”, W81º44’13.6” |
| Cocos | Gulf of Chiriquí | N7º36’56.9”, W81º42’51.2” |
| Los Frijoles (Frijol & Frijolito) | Gulf of Chiriquí | N7º39’16.6”, W81º43’40.7” |
| Canales de Afuera | Gulf of Chiriquí | N7º41’45.4”, W81º37’58.5” |
| Granito de Oro | Gulf of Chiriquí | N7º35’38.2”, W81º42’46.2” |
| Pacheca | Gulf of Panama | N8º39’46.1”, W79º03’20.6” |
| Chapera | Gulf of Panama | N8º35’31.3”, W79º01’08.6” |
| Saboga | Gulf of Panama | N8º37’42.4”, W79º03’18.6” |

Table S2: Study species – Tropical reef fish of Panama***.*** Skin microbiome samples were collected from 10 species of tropical reef fish, including representatives from a range of trophic groups and four different families (Acanthuridae, Chaetodontidae, Pomacentridae, and Serranidae).


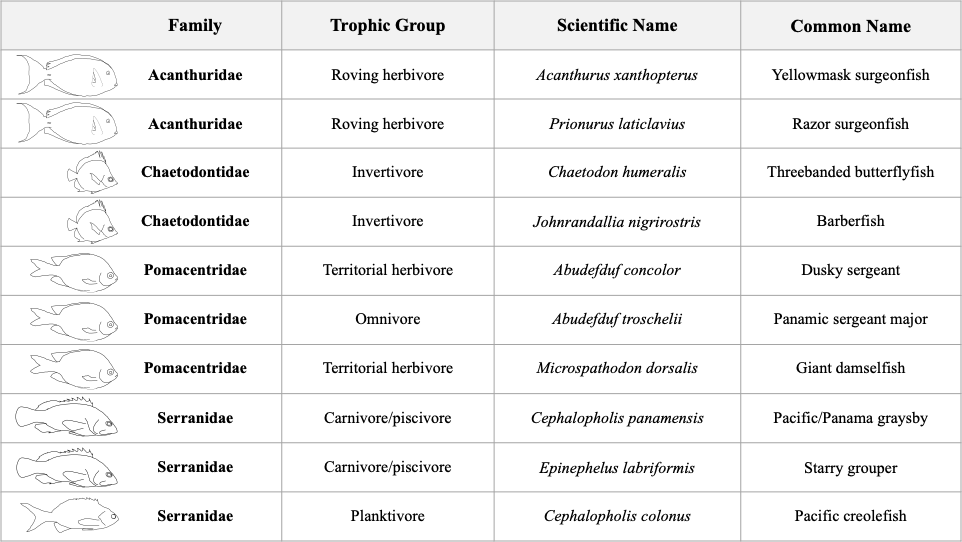


Table S3: PCR conditions for 16S rRNA gene amplification. We amplified the V4 region of the 16S ribosomal RNA gene (16S rRNA) using four sets of phased primers 515F ([CTA]GTGYCAGCMGCCGCGGTAA) and 806R ([ATC]GGACTACNVGGGTWTCTAAT) adapted for Illumina sequencing. Nucleotides in brackets ([ATC]/ [CTA]) were added one-by-one to the phased primer sets, such that 515F/806R “IL” had no added nucleotides, “ILN” had 1 (blue C/A), “IL2N” had 2 (green & blue), and “IL3N” had 3 (red, green, blue). Full sequences for both 16S rRNA and index PCR available in the metadata excel file.

*PCR Conditions*

*35 Cycles*

| 94ºC | 94ºC | 50ºC | 72ºC | 72ºC | 10ºC |
| --- | --- | --- | --- | --- | --- |
| 3 min | 45 s | 1 min | 1 min 30 s | 10 min | hold |

Table S4A: Fish Sample Sizes. We aimed to collect 10 individuals per species during each sampling season and region and sequenced nine skin swabs for each group. Initial sample sizes (total fish samples sequenced), sample sizes after trimming (removing samples with fewer than 1000 reads), and sample sizes after rarefaction to 2500 reads given by species for each gulf (*G of C* = Gulf of Chiriquí, *G of P* = Gulf of Panama).


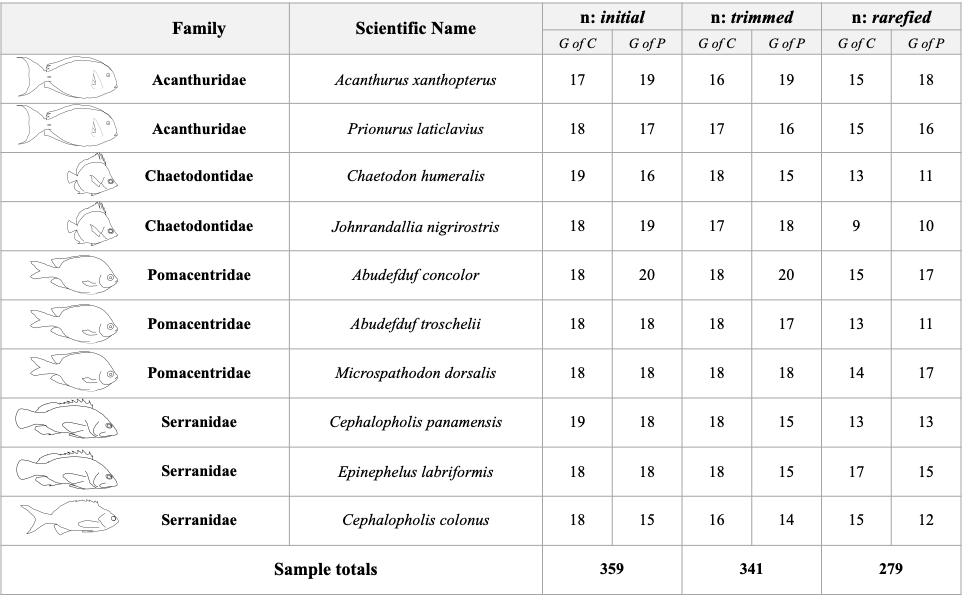


Table S4B: Water Sample Sizes. We aimed to collect ~10 water samples per region (Gulf of Chiriquí and Gulf of Panama), with ~5 samples collected per season (sampling during the wet and dry seasons). Sampling locations in each gulf matched our fish sampling sites.

Supplementary files S1 & S2: contaminant detection with decontam. The `decontam` R package was used to identify potential contaminant sequences in both the fish skin and water datasets, using the prevalence method which identifies contaminants based on their increased prevalence in control samples. These output tables include all sequences (raw_sequence), their frequency in the dataset – normalized by default so that each row in the sequence table sums to 1 (freq), their prevalence (prev), a calculated ‘p.prev’ value between 0 & 1*, and a logical TRUE/FALSE call for if the sequence is a contaminant (contaminant).

Fish samples: “Supp_file_S1_Fish_ROHR_06_contamdf.prev.csv”

Water samples: “Supp_file_S2_Water_ROHR_08_contamdf.prev.csv”

*The p.prev value is based on a comparison of how the sequence’s prevalence matches one of two models: a contaminant and a non-contaminant model, where a low score (near 0) indicates better matching with the contaminant model and a high score (near 1) means the sequence aligns more with expected distributions for non-contaminants. See Davis et al. (2018) in *Microbiome* for more details on this methodology.

# **Part 2:** *Environmental Parameters*

***
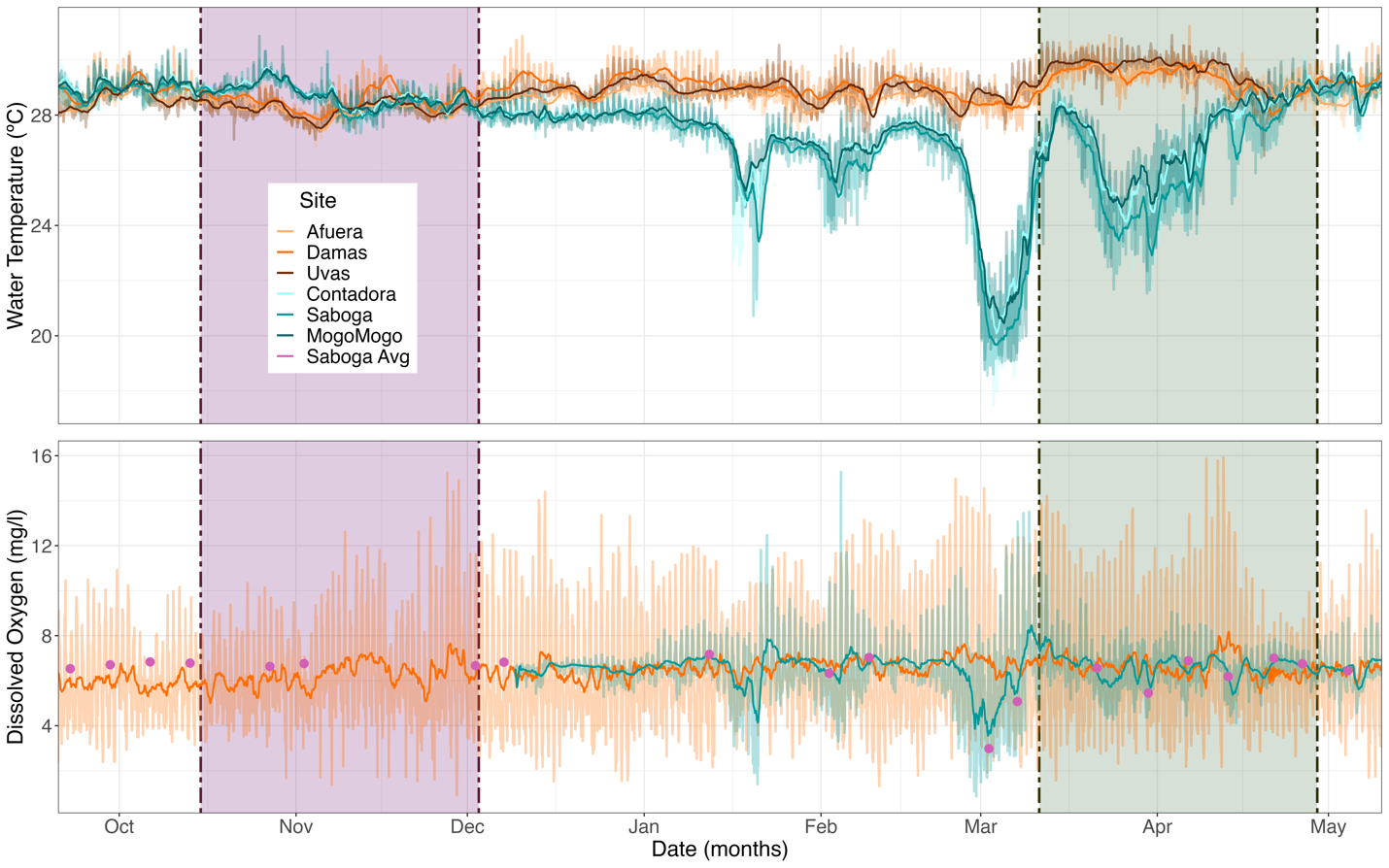
***

**A**

**B**

Figure S1: Water temperature and dissolved oxygen in Panama’s Tropical Eastern Pacific. (**A**) Temperature (ºC) and (**B**) dissolved oxygen (DO; mg/L) readings at our sampling sites in the Gulf of Chiriquí (*orange*) and Gulf of Panama (*turquoise*). Vertical shaded bars denote sampling dates during the wet (Oct-Nov; *purple*) and dry (Mar-Apr; *green*) seasons. Dry season sampling began shortly after water temperatures dropped in the Gulf of Panama, indicative of upwelling. Simple moving average (dark lines; values averaged across (**A**) 48 and (**B**) 24 hours) overlaid over raw temperature/dissolved oxygen values. (**B**) Dissolved oxygen data loggers were only deployed in the Gulf of Panama in December 2021; additional data reported from STRI’s Physical Monitoring Program, which takes weekly manual DO measurements (Saboga Avg: *purple points*).*

**Notes for Fig. S1*: We deployed loggers at fixed depths in shallow reef sites in both gulfs (Temp: 4.6-6.1 m, DO: 5.5-7.2 m avg. depth). Our loggers collected data in 15- or 30-minute intervals, and we later standardized the merged dataset to 30 minutes. Given that the DO logger for the Gulf of Panama was deployed part way through our sampling period (December 2021), we sourced additional DO data from the STRI Physical Monitoring Program (PMP), which has measured environmental parameters, including DO, weekly across the Gulf of Panama since 2017, using a manual YSI EXO 2 sonde (35). These data measure DO across a range of depths, in approximately 5-m depth intervals, from around 5 to 20 m, and multiple measurements are taken consecutively at each depth. Since our loggers were placed at fixed depths, we subset the PMP dataset to include only the shallowest measurements (binning measurements taken at 3 to 7 m depth) and calculated the average DO for each day, then plotted these points alongside our miniDOT dataset. PMP data were generally collected between 8:30 and 10:00 AM, and average values were logged as 9:00 AM when merged with our DO data. Despite different measurement techniques, the PMP and miniDOT datasets were consistent across overlapping sampling timepoints, increasing our confidence that the PMP values for October-December 2021 reasonably estimate local conditions at our Gulf of Panama sampling sites.

# **Part 3:** Alpha diversity metrics

**B**

**A**

Figure S2: Hill numbers by host species. Boxplots of the three alpha diversity Hill numbers: observed richness (q0), Shannon exponential (q1), Simpson multiplicative inverse (q3). **(A)** unrarefied, **(B)** rarefied to 2500 reads.

Table S5: Alpha diversity metrics***.*** Various metrics assessing microbial community variation within individual fish skin samples, to gage community diversity, evenness, dominance, and rarity (unrarefied & rarefied to 2,500 reads/sample). Available as two separate csv files: “*Table_S5_ROHR06_skin_alpha_div_metrics_[non_rarefied]/[rarefied2500].csv*”

Table S6: Alpha diversity pairwise comparisons between species. Adjusted *P*-values (Kruskal-Wallis; Dunn post-hoc, Bonferroni-Hochberg correction) for pairwise comparisons between **(A)** fish host species and **(B)** trophic groups for observed (q0; *top right*), Shannon (q1; *top left*), and Inverse Simpson (q2; *bottom*) alpha diversity metrics run on unrarefied dataset. Significant comparisons (*P* < 0.05) are shaded in green; smaller *P*-values correspond to darker shading.

**A**

Shannon

Observed

Inv. Simpson

Shannon

Observed

Inv. Simpson

**B**

# **Part 4:** Beta diversity metrics

Table S7: PERMANOVA results. Permutational multivariate analysis of variance (PERMANOVA) were run (10000 permutations) with three dissimilarity/distance metrics: Bray-Curtis, Jaccard, and UniFrac on (1) the whole fish skin dataset, (2) the water dataset, and (3) the species-by-species datasets. All tests were run on both unrarefied and rarefied (to 2500 reads; shaded columns) datasets. Complete results are reported for each PERMANOVA, including the model run (Model), degrees of freedom (Df), sum of squares (SumOfSqs), partial R2 (R2), pseudo-*F* statistic (F), p-value (Pr(>F)), and residual R2 (Residual R2). Significant comparisons (*P* < 0.05) are shaded in green; smaller *P*-values correspond to darker shading. For the whole fish skin dataset, we tested trophic group, species, region, and season independently, then ran a model looking at the interactions between trophic group, species, region, site within region, and season, using the strata argument to constrain permutations within trophic group, given the nestedness of species within trophic group. For the species-by-species and water datasets, we tested region, season, and the interaction between region and season. Excel file: “Table_S7_*PERMANOVA results raw.xlsx*”

***
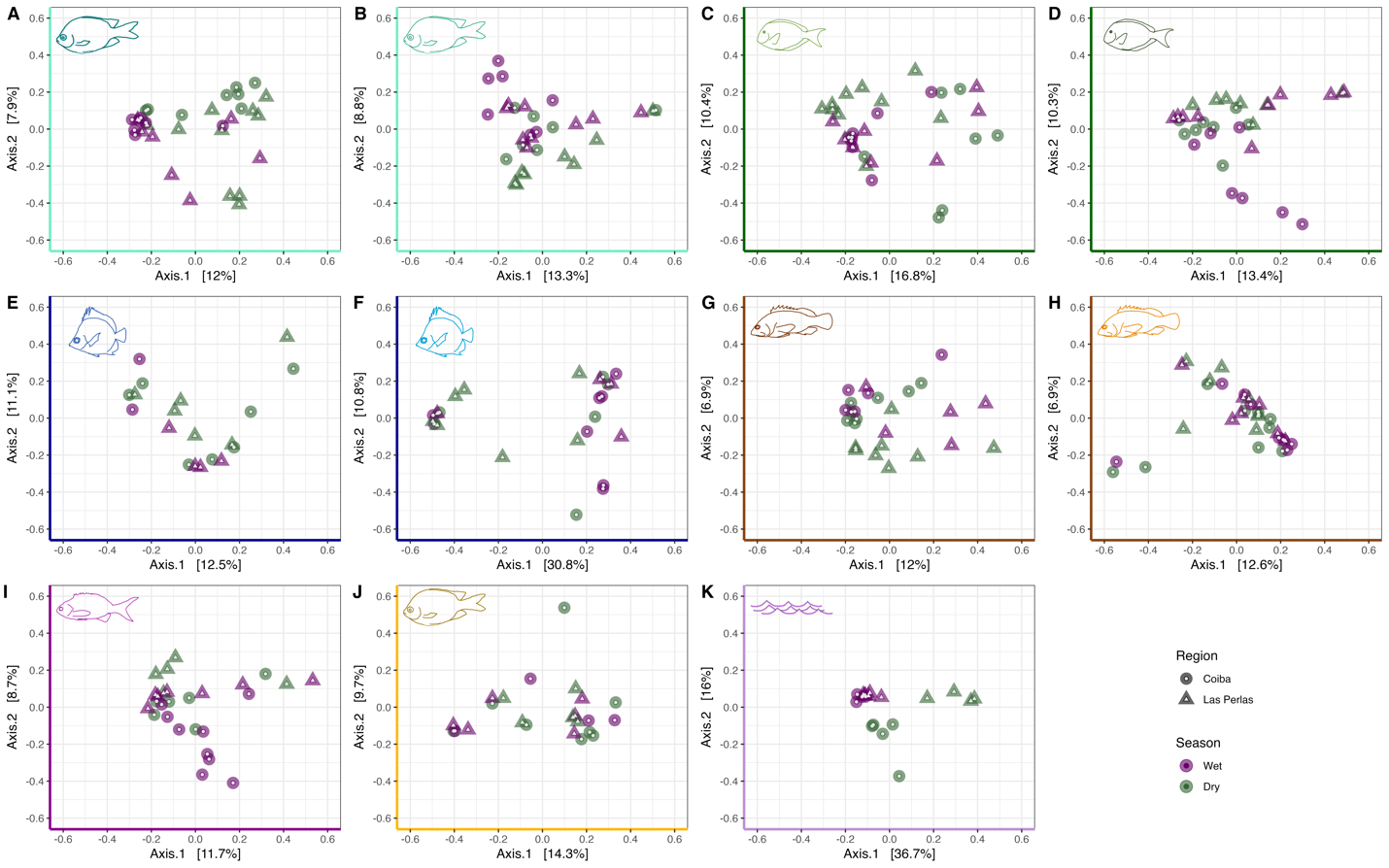
***

Figure S3: Dissimilarity between microbial communities - PCoA: rarefied to 2500 reads. PCoA plots displaying all microbiome samples included in this study, based on Bray-Curtis dissimilarities between communities. **(A – J)** Fish skin microbiomes across regions (*circle*: Gulf of Chiriquí; *triangle*: Gulf of Panama) and seasons (*purple*: non-upwelling, *green*: upwelling), split by host species. Axis colours and icons indicate trophic groups and study species, respectively (see *Fig. 1*). **(K)** Water microbiome, included for comparison. Individual points represent single fish (***n = 279***) or water (***n = 19***) samples.

******

Figure S4A: Dissimilarity between microbial communities - NMDS. NMDS plots displaying all microbiome samples, based on Bray-Curtis dissimilarities between communities. **(A – J)** Fish skin microbiomes across regions (circle: Gulf of Chiriquí; triangle: Gulf of Panama) and seasons (purple: non-upwelling, green: upwelling), split by host species. Axis colours and icons correspond to each of our study species. **(K)** Water microbiome, included for comparison. Individual points represent single fish (***n = 341***) or water (***n = 19***) samples.

******

Figure S4B: Dissimilarity between microbial communities with covariates - NMDS. NMDS plot displaying Bray-Curtis dissimilarities of all fish skin microbiome samples. Prior to generating this plot, water temperatures were averaged across 24hrs in each region, then these results were merged with the fish sample metadata (i.e., matching the average temperature from the region with the day the fish was sampled). Vectors of key predictors, filtered to show the top 50% that explained the most variance in the dataset, are overlaid on the ordination. Vector length represents the amount of variance explained (longer = greater explanatory power) by each predictor, however, the temperature vector was scaled down by ½ so it could be visualized alongside all the other vectors. Note that “Las Perlas Upwelling” is directly opposite “Temperature”, suggesting warmer (non-upwelling) temperatures are driving patterns in the lower right-hand corner while communities associated with colder, upwelling conditions in Las Perlas concentrate in the upper left-hand side.


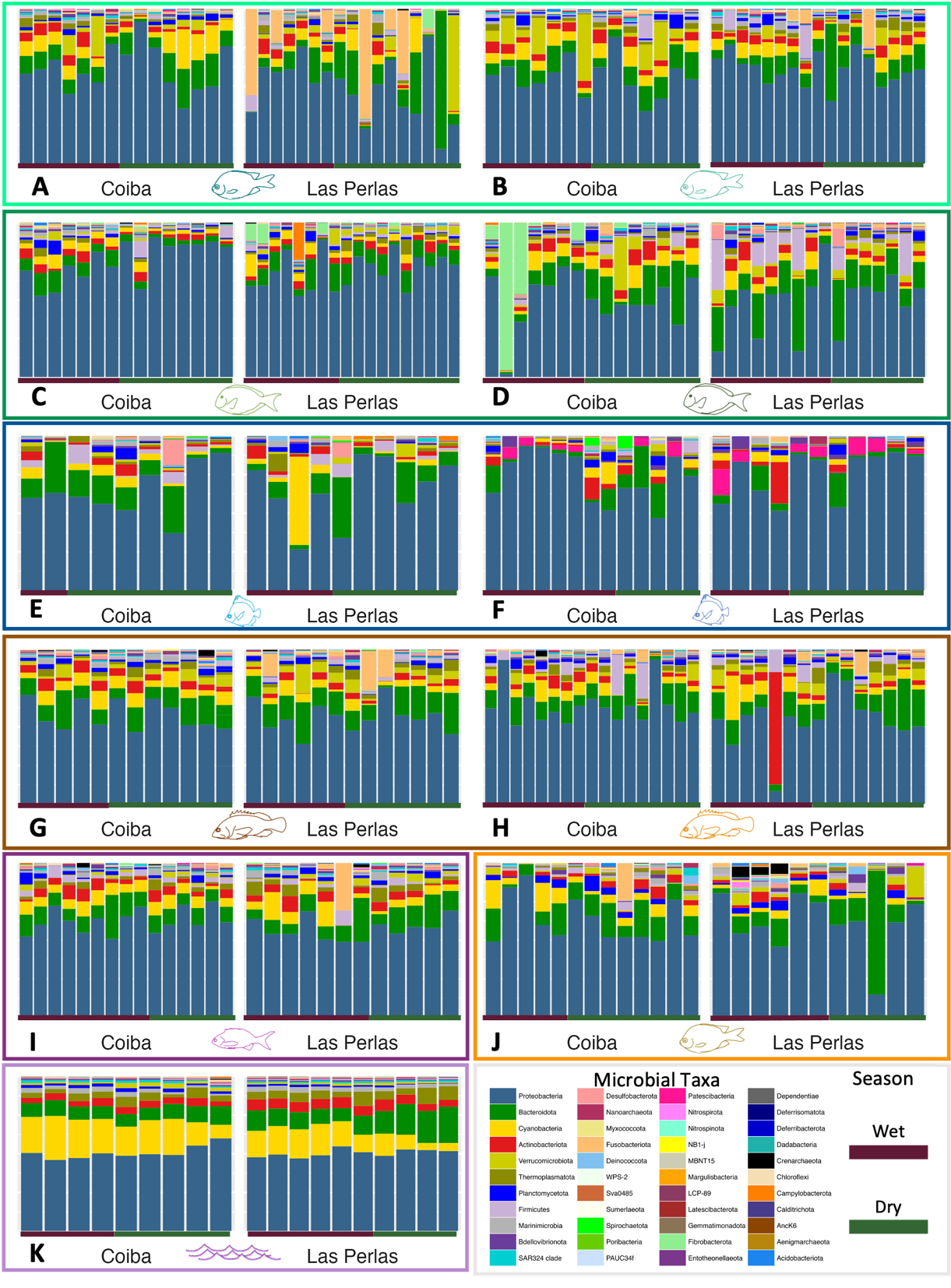


Figure S5: Relative abundances of skin microbial phyla – rarefied to 2500 reads***.*** Stacked bar plots of phylum-level microbiome composition, split by gulf (*left:* Coiba - Gulf of Chiriquí, *right:* Las Perlas - Gulf of Panama). **(A - J)** Individual bars represent a single fish. Panels arranged by trophic group (coloured boxes), fish icons denote species (see *Fig. 1*), and coloured bars along the bottom of each panel mark sampling seasons (*purple* = wet, *green* = dry). **(H)** Water microbiome community composition from the surrounding reef for comparison.

# **Part 5:** Differential abundance analyses

## MaAsLin2:

*MaAsLin2 Methods*

In addition to DESeq2, as described in the main text, we also ran MaAsLin2 on our water dataset to compare the two differential abundance methods. As for DESeq2, un-normalized counts were used for MaAsLin2, and taxa present in less than 10% of samples in each dataset were filtered out. MaAsLin2 was run with standard parameters; applying total sum scaling for normalization, log-transforming the data, running a linear model with “region_season” as a fixed effect (this column returns all the region and season combinations), and applying Benjamini-Hochberg (BH) correction for multiple comparison, setting the significance threshold at 0.05. Results from both tests for the water dataset are reported below, and DESeq2 was chosen to run on the fish dataset, given the flexibility provided by the “contrasts” argument for testing pairwise comparisons. When testing more than two groups (i.e., region and season), for MaAsLin2, we set the reference as Gulf of Panama wet season (non-upwelling) and ran pairwise comparisons between this reference and (1) Gulf of Panama dry season (upwelling), (2) Gulf of Chiriquí wet season, and (3) Gulf of Chiriquí dry season. DESeq2 was run with standard parameters, specifying the desired contrasts, and returning the log2 fold change, Wald test p-values, and adjusted p-values with Benjamini-Hochberg (BH) multiple comparison correction. The significance threshold was set to p-adj = 0.05.

*MaAsLin2 Results and Comparison with DESeq2*

For the water dataset, out of 1298 filtered taxa (49.5% of the initial 2624 taxa passed 10% filter step; *see methods*), 524 and 528 taxa were significantly differentially abundant between the Gulf of Panama wet and dry season samples for DESeq2 and MaAsLin2, respectively.. Among these, 318 (24%) were enriched (log-fold change; LFC >0) and 206 (16%) decreased in the wet (non-upwelling) relative to the dry (upwelling) season (DESeq2) while for MaAsLin2, 237 were enriched during the dry season and 291 decreased. In contrast, across gulfs during the wet season there were only 116 DA taxa identified by DESeq2 and 185 by MaAsLin2. While seasonal shifts were lesser in the Gulf of Chiriquí, there were still 350 DA taxa detected between the wet and dry seasons (DESeq2). When comparing the two gulfs during the dry season, there were 465 DA taxa (188 increased and 277 decreased in the Gulf of Panama relative to the Gulf of Chiriquí; DESeq2). The log2-fold changes in the top 25 DA taxa for each set of pairwise comparisons are plotted in Fig. S6 as well as the top 50 DA taxa for the comparison between Gulf of Panama wet and dry seasons (*Fig. S7*). Note that given the structure of MaAsLin2, we set the Gulf of Panama wet season as the reference, and thus only have data to compare with DESeq2 for Gulf of Panama wet vs. dry and Gulf of Panama wet vs. Gulf of Chiriquí wet.

Table S8: Differentially abundant taxa between regions and seasons for fish and water microbiomes. Significant DESeq2 results (p-adj < 0.05, Wald test, BH correction) reported for four key region-season comparisons: Gulf of Chiriquí (Coiba) wet vs. dry season, Gulf of Panama (Las Perlas) wet vs. dry season, Gulf of Panama wet season vs. Gulf of Chiriquí wet season, and Gulf of Panama dry season vs. Gulf of Chiriquí dry season. For fish, bold values indicate total significant DA taxa, values in parentheses are shared DA taxa between fish and water samples. The complete list of DA taxa for both fish and water samples can be found in supplementary file: “Table_S8_ALL_fish_and_water_significant_DA_taxa.csv”

|  | Gulf of Chiriquí wet vs. dry | Gulf of Panama wet vs. dry | G of Panama vs. G of Chiriquí wet | G of Panama vs. G of Chiriquí dry | Total DA taxa |
| --- | --- | --- | --- | --- | --- |
| *Abudefduf concolor* | **2** (0) | **4** (2) | **7** (0) | **4** (0) | **17** (2) |
| *Microspathodon dorsalis* | **3** (0) | **20** (13) | **4** (0) | **15** (9) | **42** (22) |
| *Acanthurus xanthopterus* | **22** (3) | **0** (0) | **14** (1) | **22** (10) | **58** (14) |
| *Prionurus laticlavius* | **14** (1) | **27** (20) | **12** (0) | **20** (14) | **73** (35) |
| *Cephalopholis panamensis* | **6** (0) | **4** (0) | **7** (2) | **1** (0) | **18** (2) |
| *Epinephelus labriformis* | **12** (0) | **4** (0) | **10** (1) | **7** (2) | **33** (3) |
| *Cephalopholis colonus* | **1** (0) | **7** (3) | **4** (0) | **5** (1) | **17** (4) |
| *Water* | **350** | **524** | **116** | **465** | **1455** |

Table S9: Significant DA taxa shared between fish and water microbiomes. All microbial taxa that shared significant DESeq2 results (p-adj < 0.05, Wald test, BH correction) between fish skin and water in one or more of four key region-season comparisons: Gulf of Chiriquí (Coiba) wet vs. dry season, Gulf of Panama (Las Perlas) wet vs. dry season, Gulf of Panama wet season vs. Gulf of Chiriquí wet season, and Gulf of Panama dry season vs. Gulf of Chiriquí dry season. The fish host species, microbial taxonomy (phylum + highest resolution assignment), ASV sequence, contrast (region-season comparison), log2-fold change (for the fish and for the water), and direction of change (T/F: were taxa enriched or depleted in the same direction for fish and water?) are reported for each shared significant DA taxa. Available as a separate csv file: “*Table_S9_shared_significant_DA_taxa_water_fish.csv*”


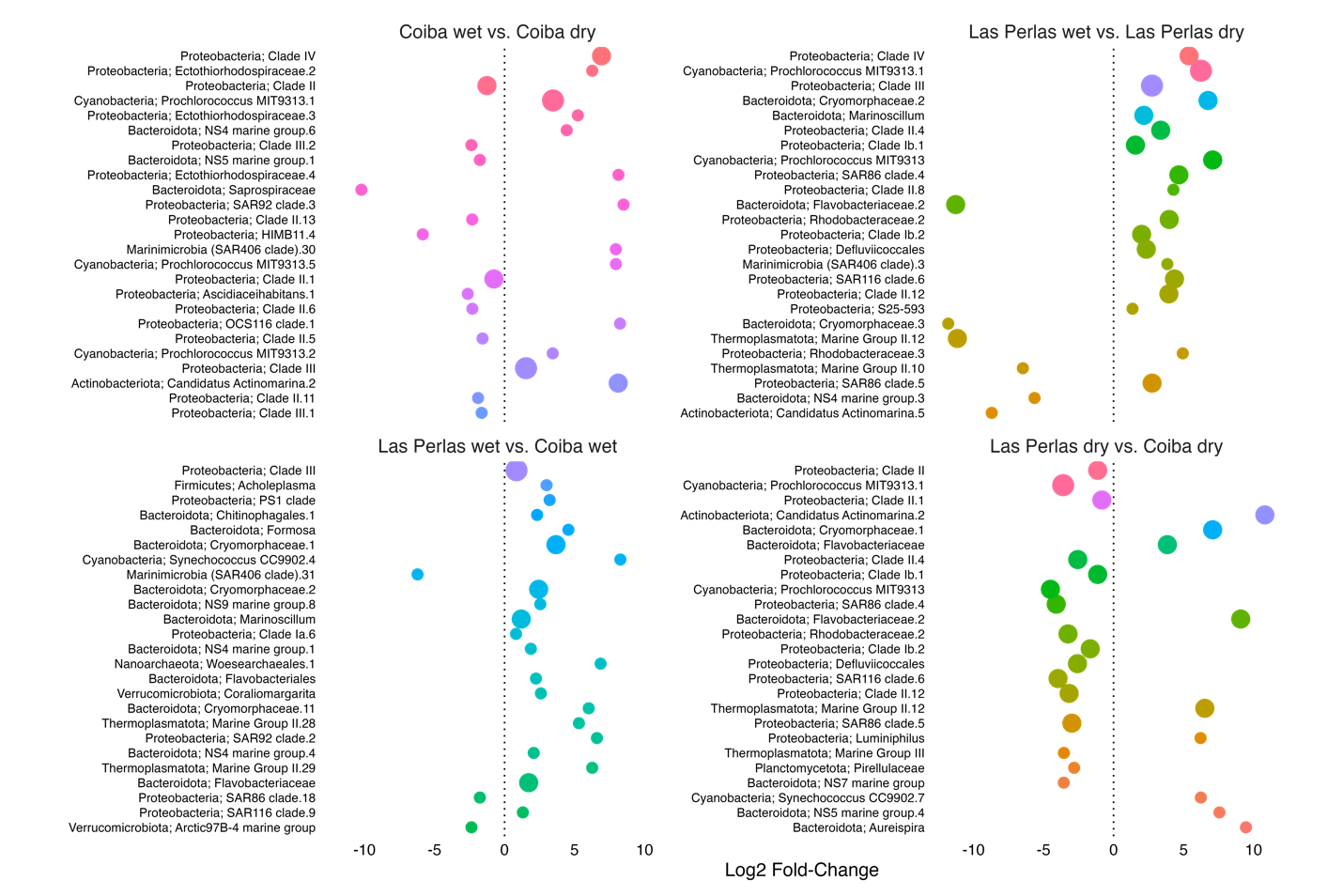


Figure S6: Water taxa differential abundance between regions and seasons. Log2-fold changes in top 25 most significantly differentially abundant taxa (DESeq2: Wald test, BH correction) per set of pairwise comparisons between water microbiome communities (e.g., Coiba – Gulf of Chiriquí - wet season vs. Coiba dry season; top left-hand panel). Lowest p-adjusted values displayed at top of plot (p-values for these taxa ranged from 5.2e-57 to 1.8e-04). Point size corresponds to the number of times each taxon was among the top 25 taxa across all four panels and colours are maintained across all panels.


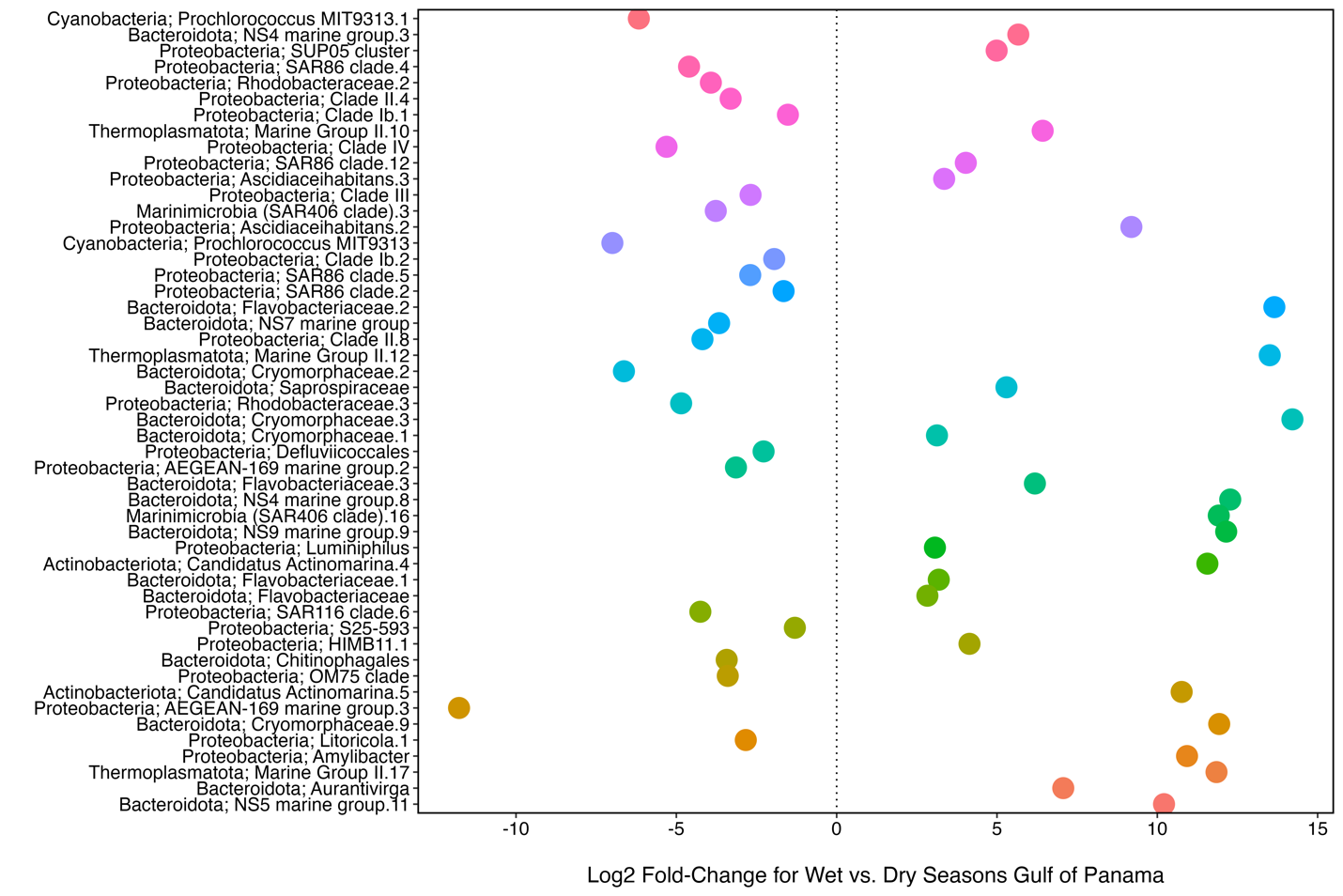


Figure S7: Water taxa differential abundance between seasons in Las Perlas. Log2-fold changes in top 50 most significantly differentially abundant taxa (DESeq2: Wald test, BH correction) in water microbiome samples between the wet and dry (upwelling) seasons in Las Perlas, Gulf of Panama. Lowest p-adjusted values displayed at top of plot (p-values for these taxa ranged from 5.2e-57 to 1.8e-04).


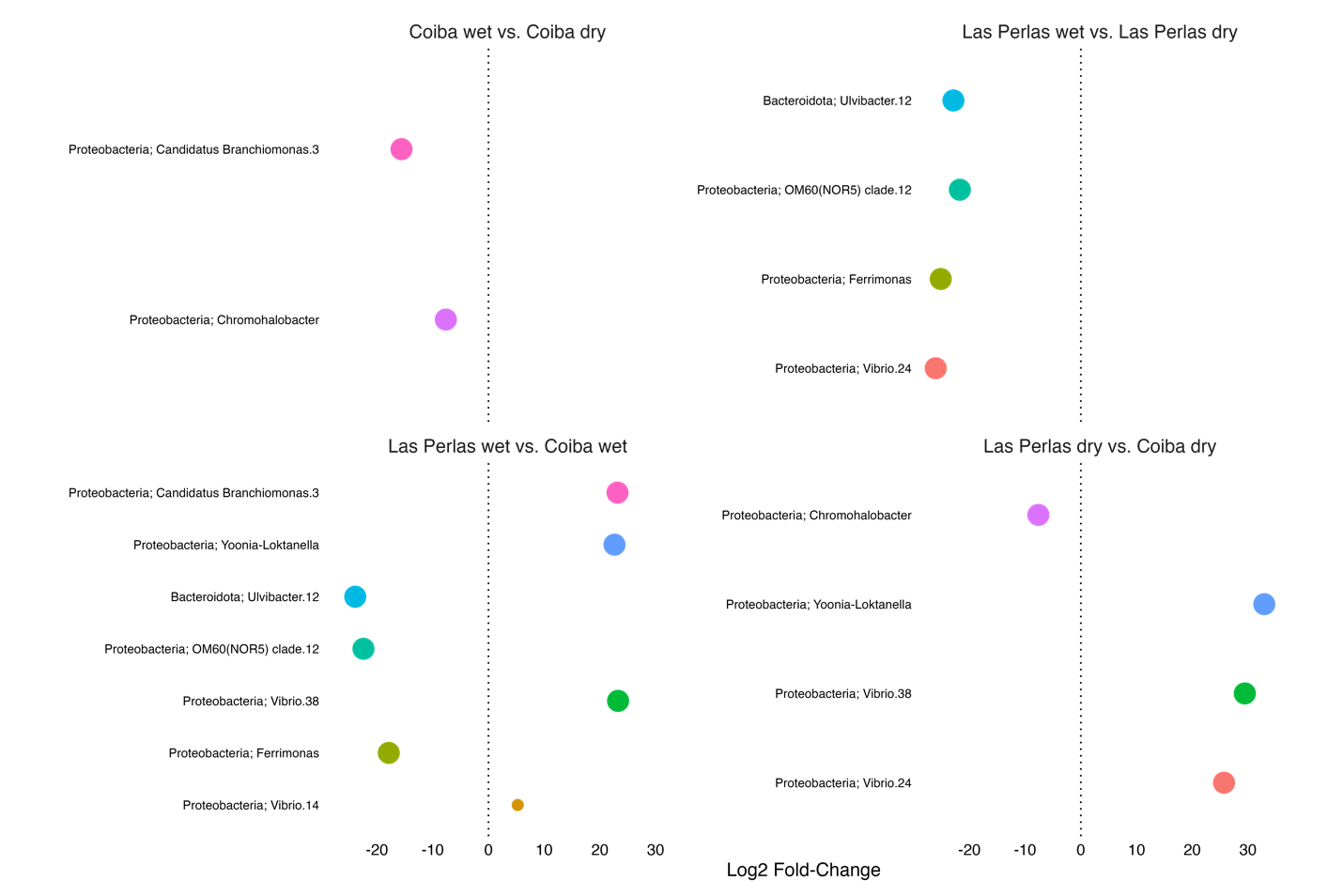


Figure S8: A. concolor taxa differential abundance in between seasons and regions. Log2-fold changes in all significantly differentially abundant taxa (DESeq2: p-adj < 0.05, Wald test, BH correction) per set of pairwise comparisons between fish microbiome communities (e.g., Coiba – Gulf of Chiriquí wet season vs. Coiba dry season; top left-hand panel). Lowest p-adjusted values displayed at top of plot. Point size corresponds to the number of times each taxon was among the significant taxa across all four panels and colours are maintained across all panels.


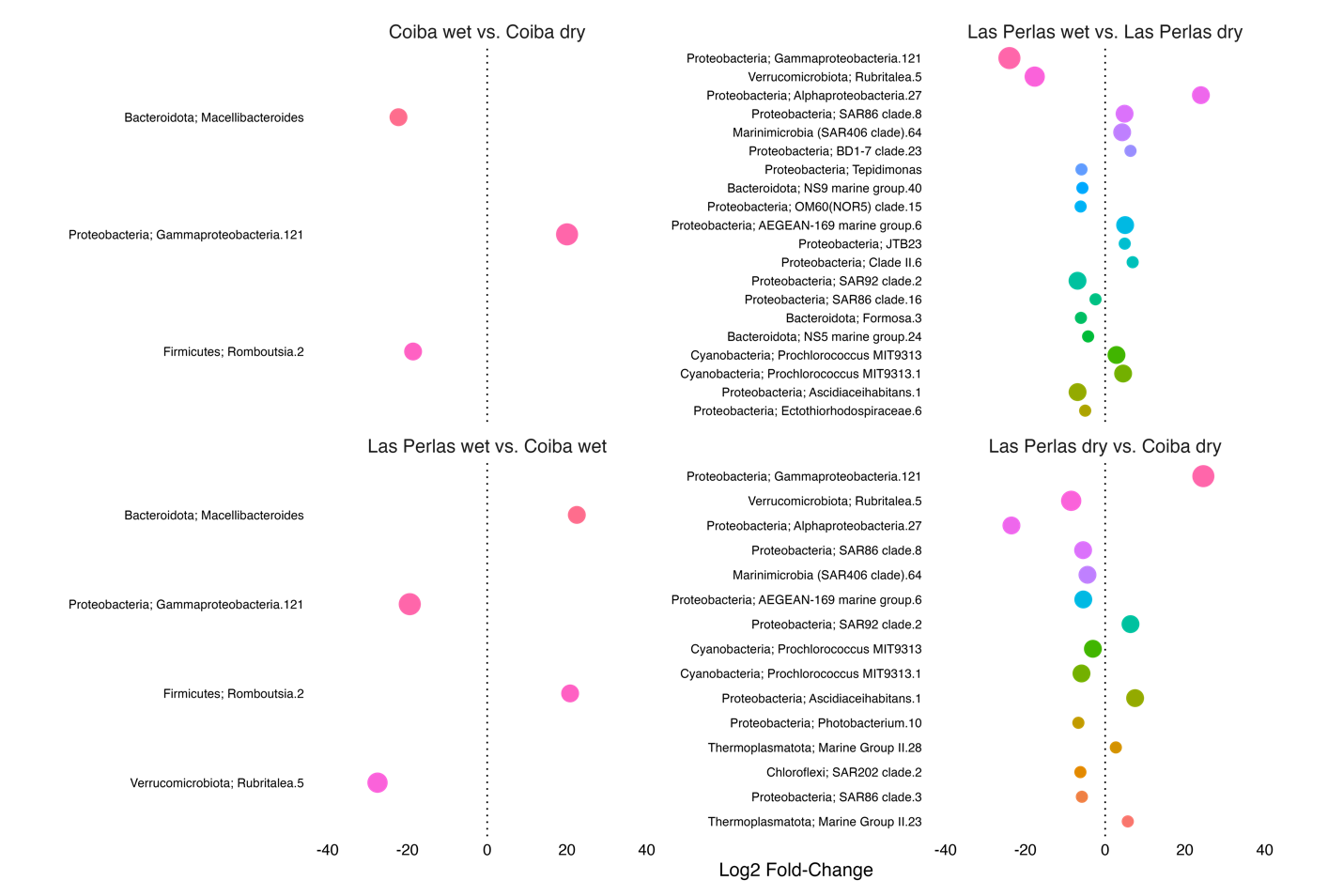


Figure S9: M. dorsalis taxa differential abundance between seasons and regions. Log2-fold changes in all significantly differentially abundant taxa (DESeq2: p-adj < 0.05, Wald test, BH correction) per set of pairwise comparisons between fish microbiome communities (e.g., Coiba – Gulf of Chiriquí wet season vs. Coiba dry season; top left-hand panel). Lowest p-adjusted values displayed at top of plot. Point size corresponds to the number of times each taxon was among the significant taxa across all four panels and colours are maintained across all panels.


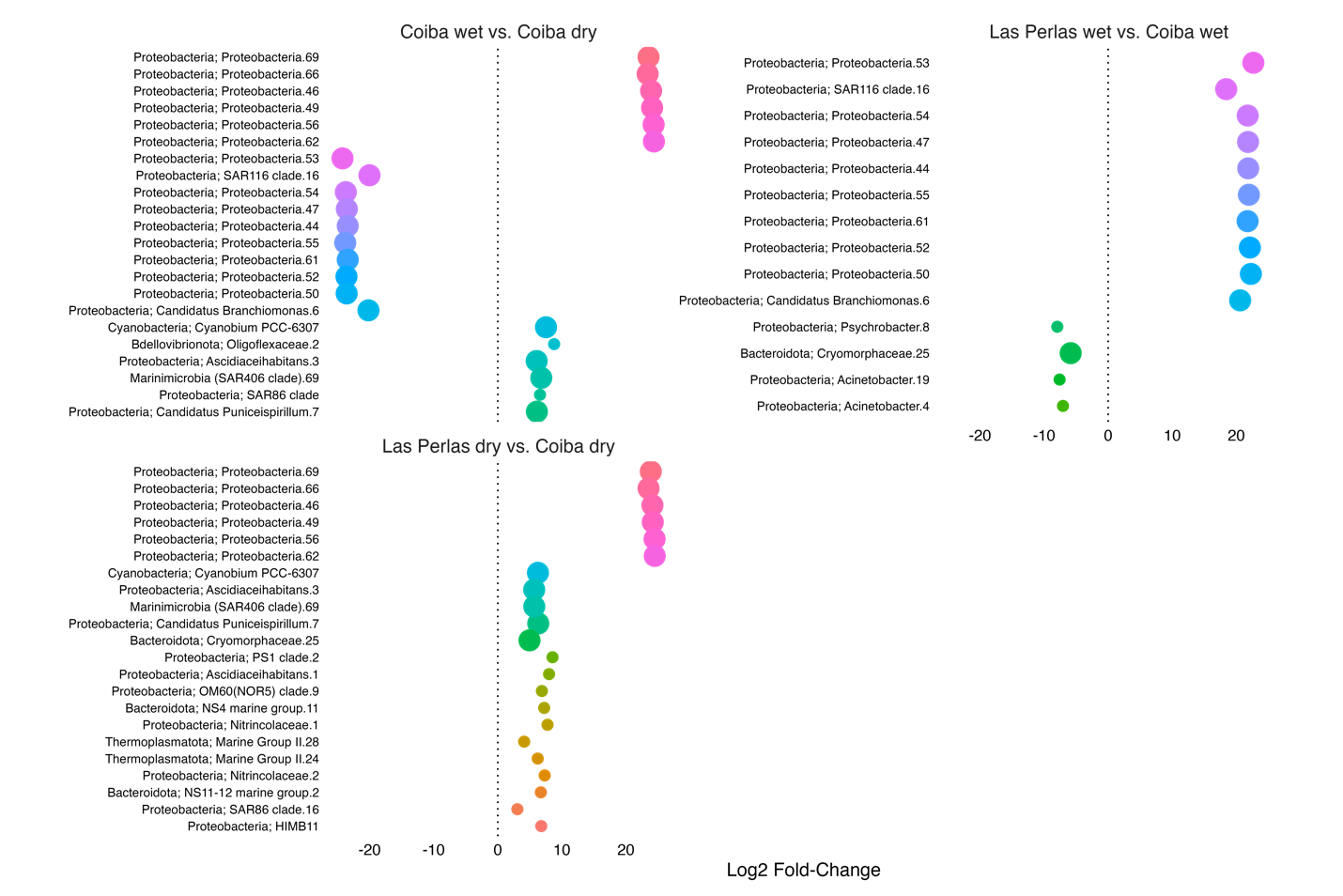


Figure S10: A. xanthopterus taxa differential abundance between seasons and regions. Log2-fold changes in all significantly differentially abundant taxa (DESeq2: p-adj < 0.05, Wald test, BH correction) per set of pairwise comparisons between fish microbiome communities (e.g., Coiba – Gulf of Chiriquí wet season vs. Coiba dry season; top left-hand panel). Lowest p-adjusted values displayed at top of plot. Point size corresponds to the number of times each taxon was among the significant taxa across all four panels and colours are maintained across all panels.


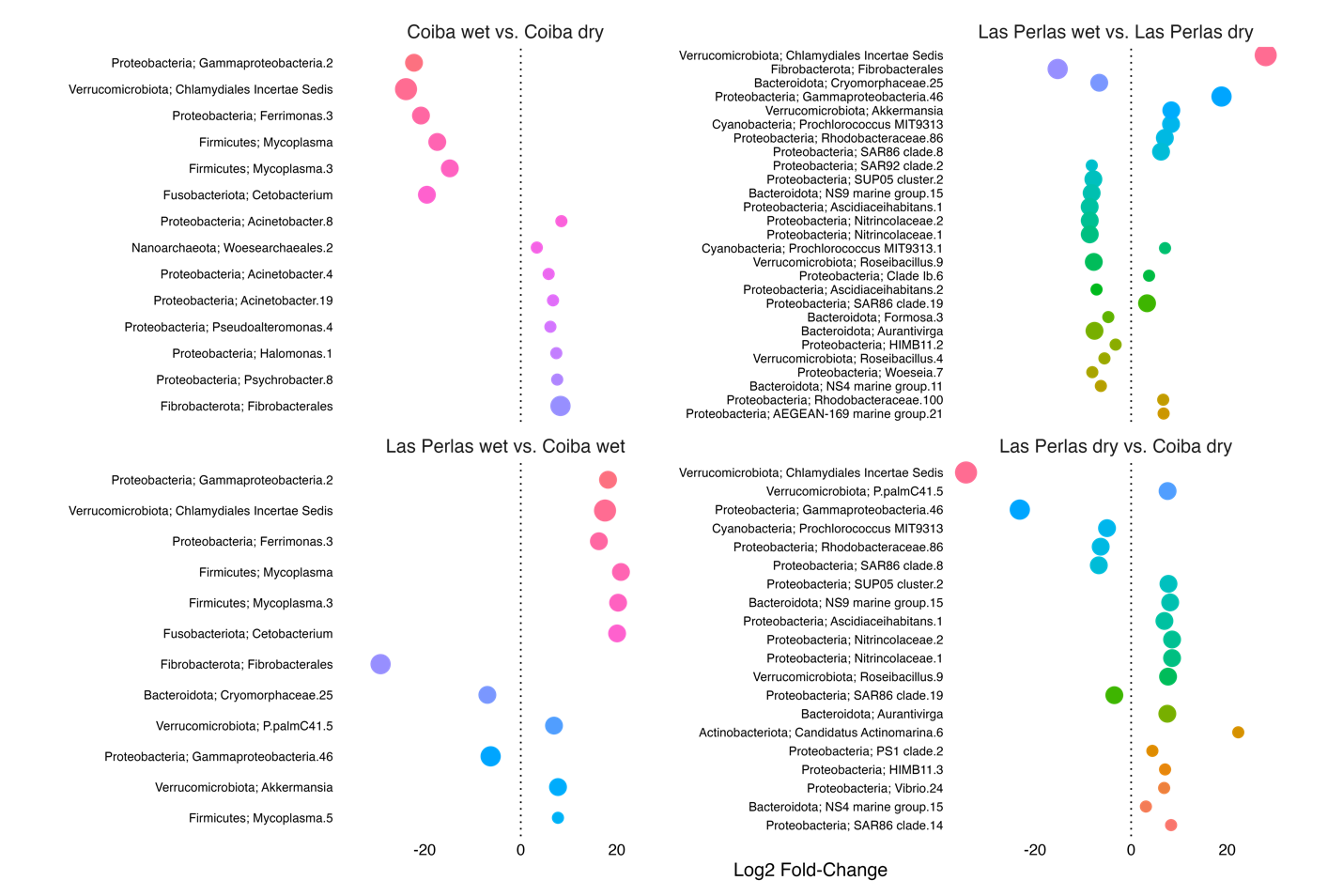


Figure S11: P. laticlavius taxa differential abundance in between seasons and regions. Log2-fold changes in all significantly differentially abundant taxa (p-adj < 0.05, Wald test, BH correction) per set of pairwise comparisons between fish microbiome communities (e.g., Coiba – Gulf of Chiriquí wet season vs. Coiba dry season; top left-hand panel). Lowest p-adjusted values displayed at top of plot. Point size corresponds to the number of times each taxon was among the significant taxa across all four panels and colours are maintained across all panels.


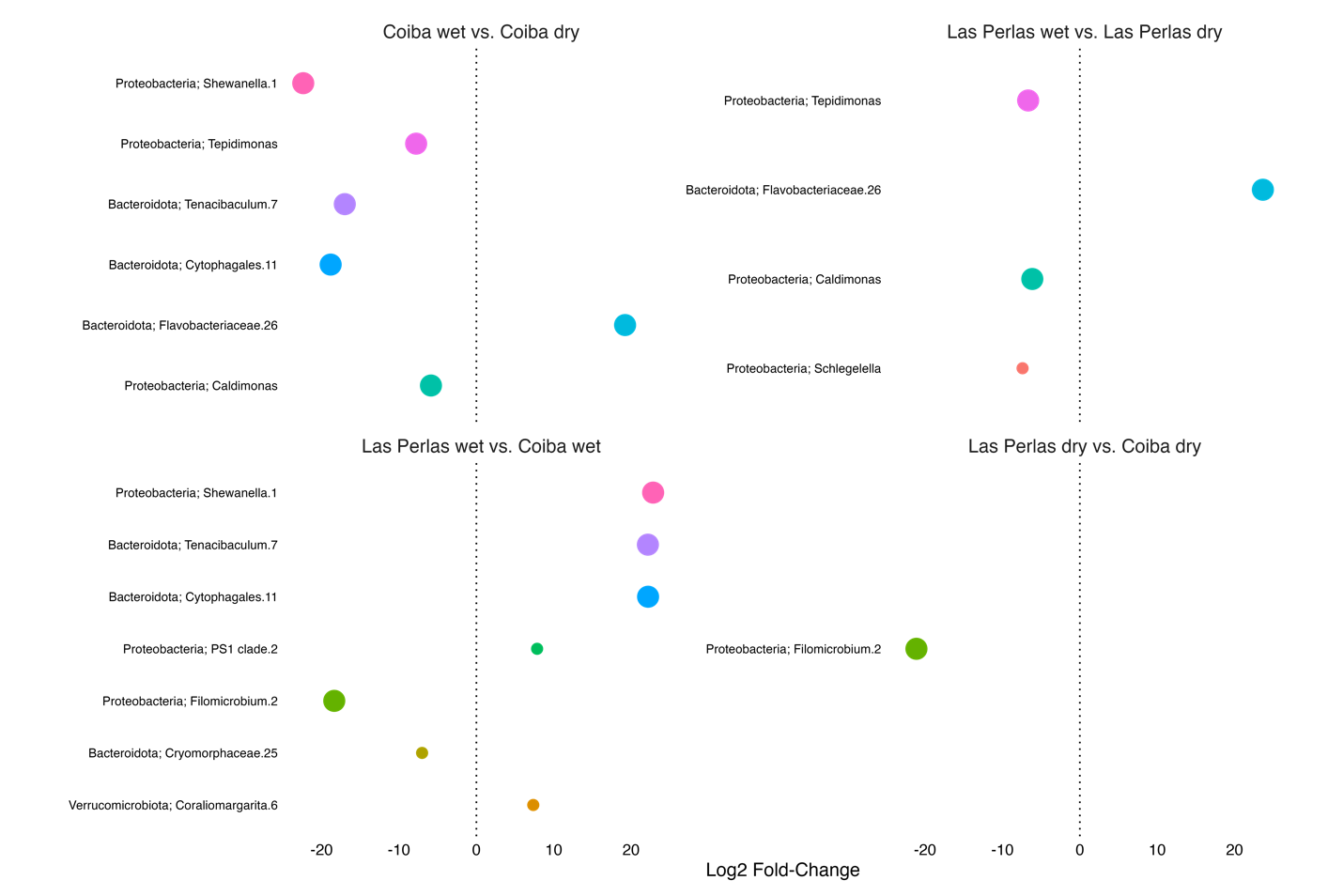


Figure S12: C. panamensis taxa differential abundance in between seasons and regions: DESeq2. Log2-fold changes in all significantly differentially abundant taxa (p-adj < 0.05, Wald test, BH correction) per set of pairwise comparisons between fish microbiome communities (e.g., Coiba – Gulf of Chiriquí wet season vs. Coiba dry season; top left-hand panel). Lowest p-adjusted values displayed at top of plot. Point size corresponds to the number of times each taxon was among the significant taxa across all four panels and colours are maintained across all panels.


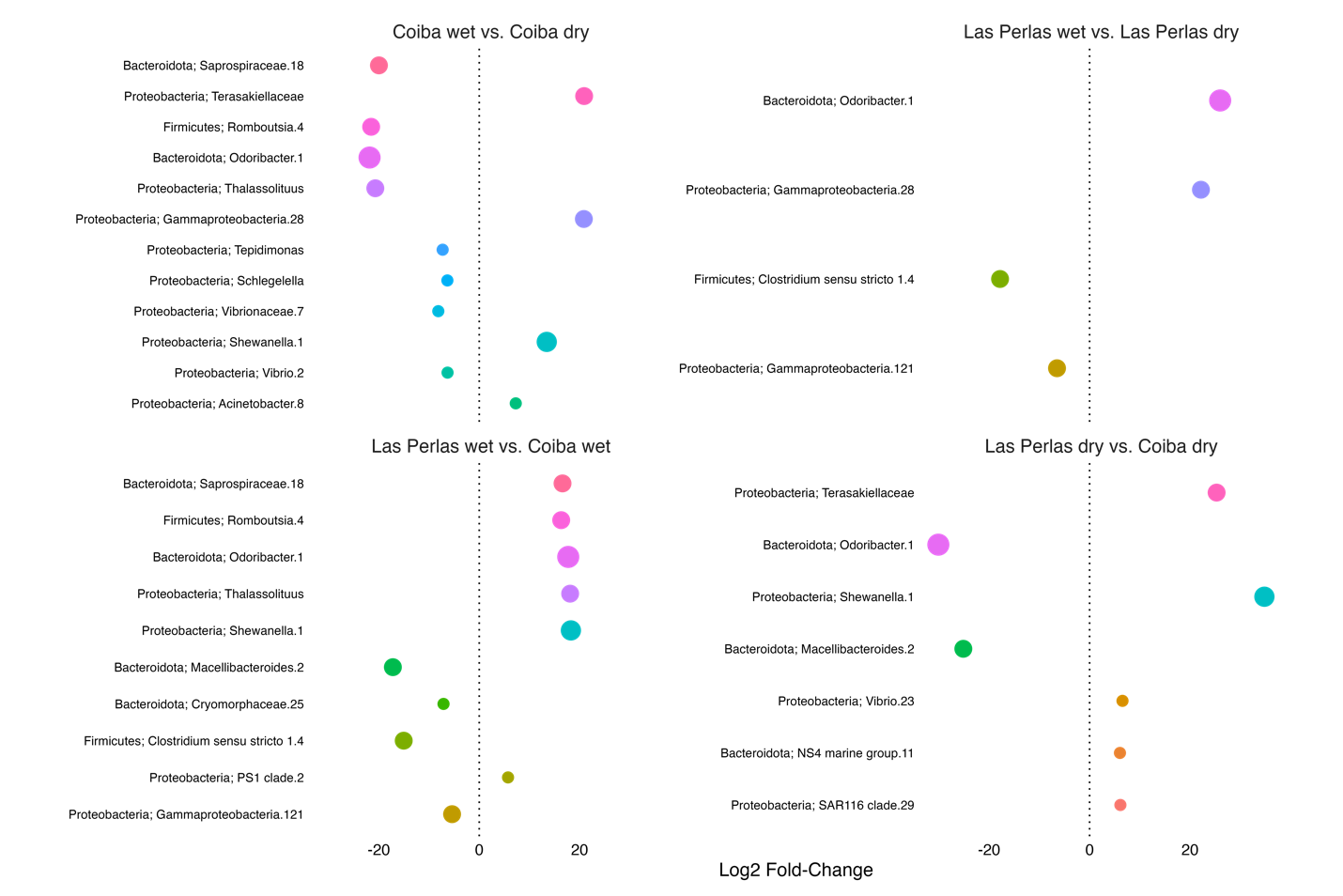


Figure S13: E. labriformis taxa differential abundance in between seasons and regions: DESeq2. Log2-fold changes in all significantly differentially abundant taxa (p-adj < 0.05, Wald test, BH correction) per set of pairwise comparisons between fish microbiome communities (e.g., Coiba – Gulf of Chiriquí wet season vs. Coiba dry season; top left-hand panel). Lowest p-adjusted values displayed at top of plot. Point size corresponds to the number of times each taxon was among the significant taxa across all four panels and colours are maintained across all panels.


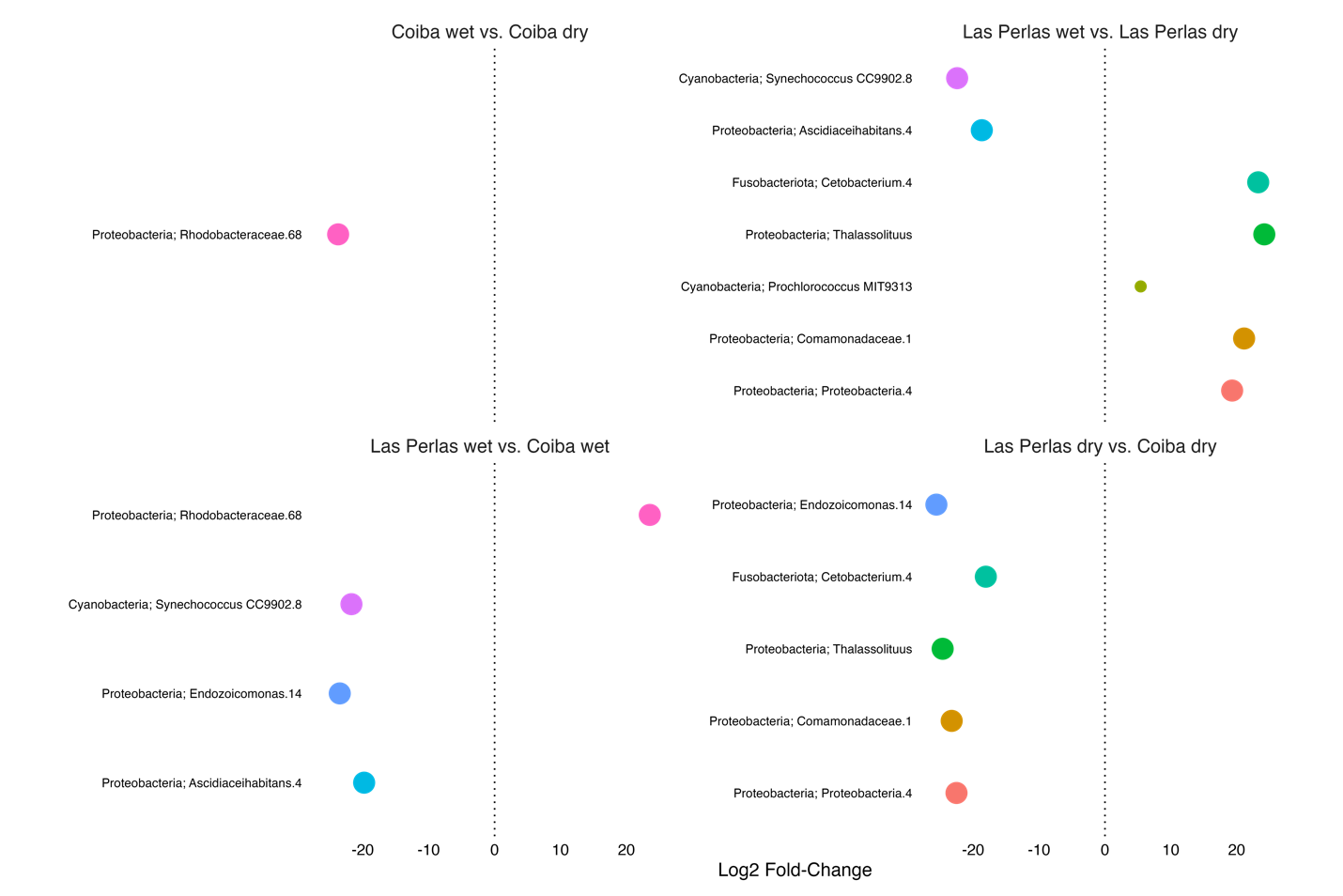


Figure S14: C. colonus taxa differential abundance in between seasons and regions: DESeq2. Log2-fold changes in all significantly differentially abundant taxa (p-adj < 0.05, Wald test, BH correction) per set of pairwise comparisons between fish microbiome communities (e.g., Coiba – Gulf of Chiriquí wet season vs. Coiba dry season; top left-hand panel). Lowest p-adjusted values displayed at top of plot. Point size corresponds to the number of times each taxon was among the significant taxa across all four panels and colours are maintained across all panels.

Figure S15: Comparison of changes in DA taxa between fish and water samples. For significant DA taxa that are shared between the fish and water datasets, log2-fold changes are plotted against each other (x = water, y = fish) to assess whether the direction and magnitude of change matches between environmental and host-associated communities. Panels are split vertically by region-season comparison (Left: Gulf of Panama - Las Perlas non-upwelling (wet) vs. Las Perlas upwelling (dry), Right: Gulf of Panama - Las Perlas upwelling (dry) vs. Gulf of Chiriquí - Coiba upwelling (dry)) and horizontally by host species (top to bottom: *Acanthurus xanthopterus*, *Microspathodon dorsalis*, *Prionurus laticlavius*; three herbivorous fishes). The dashed line represents the expected 1-to-1 correlation if changes in fish ASVs perfectly match changes in water ASVs. The blue line fits a linear model (geom_smooth( method = “lm”)) to the data, and sample sizes (n), correlation coefficient (r), and p-values are reported for each plot (within-group Pearson’s correlations). Prior to calculating statistics and plotting, the data were filtered to include only fish that shared at least 4 ASVs with water in at least one region-season comparison, excluding host species and region-season comparisons with too few points.

# **Part 6:** Core microbiome analyses

## Indicator Analysis:

We ran an Indicator Analysis (Dufrene-Legendre Indicator Species Analysis) using the indval function from `labdsv` package (v 2.1-2), following the methods outlined in Clever et al. 2022 to search for a core fish skin microbiome (adjusting p-value thresholds to 0.05). This method calculates an indicator value for each ASV and incorporates both relative frequency and abundance of ASVs in the metric. To run this analysis, we merged the unrarefied fish and water datasets, then assigned groups to each sample type for comparisons. This was done in one of two ways: first, grouping all fish together, to establish a ‘core’ of coral reef fish skin host-associated microbes vs. environmental (water) microbes. Second, grouping by host species and performing 1-to-1 comparisons between each fish species and the water samples (10 pairwise comparisons), to establish a core microbiome for each host species. ASV taxonomic assignments are reported to the genus level: in cases where genus-level identification was unavailable, the next-lowest taxonomic resolution is provided and the level is denoted with a letter code (e.g. p_Proteobacteria: phylum-level resolution).

The first test (all fish) returned 12 ‘core’ fish ASVs passing a p-value threshold of 0.05^[[1]](#footnote-1)^ (*Fig. S16*). All but one of these fish ASVs are from the phylum Proteobacteria (the other being Deinococcota), and break into classes: Alphaproteobacteria (4), Gammaproteobacteria (7), and Deinococci (1). These 12 ASVs could be considered host-associated generalist taxa: they are systematically found in all the reef fish species’ skin microbiomes, although they range in abundance and frequency across individuals. For instance, core ASV 785, a *Pseudomonas* bacterium, is found in 313 samples (out of 360 in the combined fish + water dataset), while ASV 1076, belonging to the genus *Tepidimonas*, is detected in 106 samples.

The second test (species-by-species) returned a total of 361 ASVs, of which 209 were unique (‘core’ ASVs were often found across multiple host species). Fish ranged in the number of ‘core’ ASVs they harboured, with *E. labriformis* (a grouper) topping the charts at 73, while *C. humeralis* (a butterflyfish) was at the bottom at 14^[[2]](#footnote-2)^. There were also many shared ASVs between species: 6 were shared across all species (a subset of the ones found in the test above for the ‘all fish’ core microbiome^[[3]](#footnote-3)^), then a handful are shared across 4-9 species (1-3 ASVs in each of these groupings), and more begin to be shared between 2 (n = 20) or 3 (n = 7) fish. 164 ASVs are ‘core’ members for only one fish species.

Table S10: Species-by-species core microbiomes. Core taxa (Indicator Analysis) associated with each fish host species. For each host species (fish_species): ASV IDs (ASV), indicator values (indval), p-values (pval & padj), frequency of occurrence in that species’ samples (freq), and genus-level taxonomy of the microbial taxa are reported. Available as a separate csv file: “*Table_S10_IndVal_CORE_species_vs_water.csv*”

Figure S16: Core microbial taxa and their distribution across host species. Relative abundances of the 12 ‘core’ taxa identified across all fish skin microbiomes. Core microbiomes were defined using an Indicator Analysis and comparing fish versus water microbiome communities.

1. When we apply a Bonferroni correction for multiple testing, given the thousands of ASVs contained in these samples and the default value of 1000 iterations for the indval function, all p-adjusted values are = 1, making none of these statistically significant. [↑](#footnote-ref-1)
2. These results are likely skewed by the initial microbiome richness: the patterns of numbers of core ASVs closely match the alpha-diversity patterns we observed earlier, where herbivores and carnivores had much higher observed richness than the corallivorous butterflyfishes. [↑](#footnote-ref-2)
3. In this test, we do not recover all 12 ASVs from the ‘all fish’ comparison because of the filtration thresholds applied: the same cutoffs on a larger vs. smaller dataset will have slightly more/fewer ASVs that pass the threshold. [↑](#footnote-ref-3)
